# Relationship between brain iron dynamics and blood-brain barrier function during childhood: a quantitative magnetic resonance imaging study

**DOI:** 10.1186/s12987-023-00464-x

**Published:** 2023-08-17

**Authors:** Yuto Uchida, Hirohito Kan, Gen Furukawa, Kengo Onda, Keita Sakurai, Koji Takada, Noriyuki Matsukawa, Kenichi Oishi

**Affiliations:** 1 Department of Radiology and Radiological Science, Johns Hopkins University School of Medicine, 208 Traylor Building, 720 Rutland Avenue, Baltimore, MD 21205 USA; 2 Department of Neurology, Nagoya City University Graduate School of Medical Sciences, 1, Kawasumi, Mizuho-cho, Mizuho-ku, Nagoya, 467-8601 Aichi Japan; 3 Department of Integrated Health Sciences, Nagoya University Graduate School of Medicine, 1- 1-20, Daiko-Minami, Higashi-ku, Nagoya, 461-8673 Aichi Japan; 4 Department of Pediatrics, Fujita Health University School of Medicine, 1-98, Kutsukake-cho, Dengakugakubo, Toyoake, 470-1192 Aichi Japan; 5 Department of Radiology, National Center for Geriatrics and Gerontology, Morioka-cho, Obu, 474-8511 Aichi Japan; 6 Department of Neurology, Toyokawa City Hospital, 23, Noji, Yawata-cho, Toyokawa, 442-0857 Aichi Japan; 7 The Richman Family Precision Medicine Center of Excellence in Alzheimer’s Disease, Baltimore, MD 21224 USA

**Keywords:** Blood–brain barrier, Diffusion-prepared pseudo-continuous arterial spin labeling, Magnetic resonance imaging, Pediatric brain, Quantitative susceptibility mapping

## Abstract

**Background:**

Mounting evidence suggests that the blood-brain barrier (BBB) plays an important role in the regulation of brain iron homeostasis in normal brain development, but these imaging profiles remain to be elucidated. We aimed to establish a relationship between brain iron dynamics and BBB function during childhood using a combined quantitative magnetic resonance imaging (MRI) to depict both physiological systems along developmental trajectories.

**Methods:**

In this single-center prospective study, consecutive outpatients, 2–180 months of age, who underwent brain MRI (3.0-T scanner; Ingenia; Philips) between January 2020 and January 2021, were included. Children with histories of preterm birth or birth defects, abnormalities on MRI, and diagnoses that included neurological diseases during follow-up examinations through December 2022 were excluded. In addition to clinical MRI, quantitative susceptibility mapping (QSM; iron deposition measure) and diffusion-prepared pseudo-continuous arterial spin labeling (DP-pCASL; BBB function measure) were acquired. Atlas-based analyses for QSM and DP-pCASL were performed to investigate developmental trajectories of regional brain iron deposition and BBB function and their relationships.

**Results:**

A total of 78 children (mean age, 73.8 months ± 61.5 [SD]; 43 boys) were evaluated. Rapid magnetic susceptibility progression in the brain (Δsusceptibility value) was observed during the first two years (globus pallidus, 1.26 ± 0.18 [× 10^− 3^ ppm/month]; substantia nigra, 0.68 ± 0.16; thalamus, 0.15 ± 0.04). The scattergram between the Δsusceptibility value and the water exchange rate across the BBB (*k*_*w*_) divided by the cerebral blood flow was well fitted to the sigmoidal curve model, whose inflection point differed among each deep gray-matter nucleus (globus pallidus, 2.96–3.03 [mL/100 g]^−1^; substantia nigra, 3.12–3.15; thalamus, 3.64–3.67) in accordance with the regional heterogeneity of brain iron accumulation.

**Conclusions:**

The combined quantitative MRI study of QSM and DP-pCASL for pediatric brains demonstrated the relationship between brain iron dynamics and BBB function during childhood.

**Trial registration:**

UMIN Clinical Trials Registry identifier: UMIN000039047, registered January 6, 2020.

**Supplementary Information:**

The online version contains supplementary material available at 10.1186/s12987-023-00464-x.

## Introduction

Iron is an essential mineral for normal brain development and function, as it is involved in oxygen transportation, myelin production, and neurotransmitter synthesis and metabolism [1]. The transport of iron into the brain tissue is upregulated during early development, reflecting the great demand of iron for oligodendrocyte maturation and myelination [2, 3]. Maintaining adequate iron concentration in the brain tissue is important because abnormal brain iron homeostasis from either deficiency or overload can cause common neurological impairments [4], such as neurobehavioral dysfunction in the former [5] and neurodegenerative diseases in the latter [1]. As a physiological cellular system across the blood-brain barrier (BBB), brain iron uptake is regulated by the expression of the transferrin receptor 1 on endothelial cells [1].

It has been well known that some anatomical regions, such as the globus pallidus and substantial nigra, are more prone to iron accumulation than others [6], but the mechanism of this regional heterogeneity remains unknown. Importantly, some molecular biological studies have shown that there is a close relationship between excessive iron deposition and the upregulated expression of aquaporin-4 (AQP4) [7–10]. Considering the key function of the BBB-related water exchange flow via AQP4 to maintain the brain osmotic homeostasis [9], a combined assessment of brain iron dynamics and BBB function is relevant to elucidate the interaction between both physiological systems along developmental trajectories.

Quantitative susceptibility mapping (QSM) is recognized as a unique method with which to quantify brain iron deposition, especially in the deep gray-matter nuclei [11, 12]. To date, the magnetic susceptibility trajectories of the basal ganglia plotted against age have been precisely assessed by QSM [13], in which the first two years following birth were the most dynamic phase of postnatal brain development [14–16]. Further, non-contrast magnetic resonance imaging (MRI)-based BBB imaging techniques, such as diffusion-prepared pseudo-continuous arterial spin labeling (DP-pCASL) [17], T2-based arterial spin labeling [18], BBB-filtered-exchange imaging [19], and water extraction with phase contrast arterial spin tagging [20], have been recently developed and found to be feasible in clinical settings because of their non-invasive nature and no requirement for contrast administration or catheterization [21].

In the present study, we hypothesized that rapid magnetic susceptibility progression in the basal ganglia would be associated with activated BBB function during the first few years of life. This study aimed to establish a relationship between brain iron dynamics and BBB function during childhood.

## Methods

### Study design and participants

Following approval of this single-center prospective study (UMIN000039047) by the local ethics committee (No. 2019-12-83), written, informed consent was obtained from the parents or guardians of the participants.

Study participants comprised consecutive outpatients, 2–180 months of age, who underwent brain MRI between January 2020 and January 2021 on suspicion of or for exclusion of intracranial abnormalities. Children whose parents or guardians consented to participate in this study were included. After acquiring QSM and DP-pCASL in addition to clinical MRI, the images were visually inspected (H.K., with 12 years of brain MRI acquisition experience) for quality control and were not included if inappropriate for subsequent analyses. The exclusion criteria were a history of preterm birth or birth defects, visible abnormalities on brain MRI, and diagnoses that included neurological diseases during follow-up examinations through December 2022. Eligible participants were stratified into three groups according to age in months: 2–24 months; 25–99 months; and 100–180 months.

### MRI acquisition and processing

MRI was conducted on a 3.0-T scanner (Ingenia; Philips Healthcare, Best, the Netherlands) equipped with a 32-channel head coil. When motion artifacts interfered with the MRI acquisition, midazolam [22], from 0.02 mg/kg to 0.06 mg/kg, via an intravenous route was administered for sedation by experienced neonatologists. In addition to clinical scans, a three-dimensional T1-fast field echo (FFE) sequence, which served multi-echo magnitude and phase images, was run using the following parameters: echo time (TE), 3.8–45.6 ms with 3.8 ms interval; number of echoes, 12; repetition time (TR), 52.4 ms; flip angle (FA), 15°; field of view (FOV), 192 × 192 mm^2^; matrix, 192 × 192; and 144 slices covering the whole brain, yielding an iso-voxel resolution of 1 mm^3^. The scan time for the T1-FFE sequence was 6 min 56 s. For the reconstruction of QSM, Laplacian-based phase unwrapping was applied to the multi-echo phase images [23]. Background field removal was performed using the sophisticated harmonic artifact reduction for phase data with varying kernel sizes [24]. Weighted averaging was performed on the local fields of each TE, based on the T2* map estimated from the magnitude images [25]. Then, the susceptibility map was generated from the local field map using the improved sparse linear equations and least squares method [26]. The mean susceptibility value of the cerebrospinal fluid in the lateral ventricles was defined as a zero reference, given that it was essentially water and contained negligible iron [27, 28].

To evaluate the BBB water exchange rate as the *k*_*w*_ index, the DP-pCASL sequence with a three-dimensional gradient-and-spin-echo readout was run using the following parameters: TE, 10.3 ms; TR, 4100 ms; FOV, 224 × 224 mm^2^; matrix, 64 × 64; 21 slices (zipped to 42 slices) covering the whole brain; resolution, 3.5 × 3.5 × 7.0 mm^3^ (zipped to 3.5 × 3.5 × 3.5 mm^3^); labeling duration, 1800 ms; echo planar imaging factor, 13; echo train length, 19; b values, 0 and 49.3 s/mm^2^; post labeling delay (PLD), 1800 ms; number of signal averages, 2/5/5 for the proton density/control/label images; centric ordering and optimized timing of background suppression for gray matter [29]. The proton density image was acquired for the estimation of the equilibrium magnetization in arterial blood to calculate cerebral blood flow (CBF). The scan time for the DP-pCASL sequence was 7 min 22 s. For the creation of *k*_*w*_ maps, the subtraction images were extracted from the control/label images denoised by the Marchenko–Pastur principal component analysis to improve the accuracy of the *k*_*w*_ estimation [30]. Thresholds of mean ± SD for signals during the signal-averaging process for the control/label images were applied to reduce image artifacts due to phase-incoherence. As the capillary compartment has over 100 times higher diffusivity than the tissue compartment [17], the signal contribution of the capillary compartment could be differentiated from that of the brain tissue using *b* values of 0 and 49.3 s/mm^2^.

### Calculation for *k*_*w*_ and *k*_*w*_ /CBF values

The *k*_*w*_ values were extracted based on the single-pass approximation model between the capillary fraction and *k*_*w*_ values over a range of 0–200 min^− 1^, using a look-up table approach with the following parameters: arterial transit time (ATT), 1800 ms; R1 of arterial blood, 0.6 s^− 1^; labeling efficiency, 85%; and brain–blood partition coefficient, 0.9 mL/g [21]. The ATT was fixed to 1800 ms in accordance with a previous study, which demonstrated the same ATT in children as in adults [31]. The T1 of the deep gray matter was calculated from each participant’s age using biexponential model: 501.5 × exp (–0.2827 × age) + 1156 × exp (–0.0007409 × age)/1000 [32]. The CBF values were also calculated using the signal intensity of the subtraction images with *b* value of 0 s/mm^2^ [33]. Here, the *k*_*w*_ value is defined as the ratio of the capillary permeability surface-area product of water (*PS*_*w*_) to capillary volume (*V*_*c*_):

$${k}_{w}={PS}_{w}/{V}_{c}$$

According to the Renkin-Crone Eqs. [34, 35], *PS*_*w*_ is defined as follows:

$${PS}_{w}=-\text{l}\text{n}(1-{E}_{w})\times CBF$$

where *E*_*w*_ is the water extraction ratio between the capillary and brain tissue compartments. Therefore, the *k*_*w*_/CBF value can be represented by the following equation:

$${k}_{w}/CBF=-\text{l}\text{n}(1-{E}_{w})/{V}_{c}$$

The left side is the water exchange rate across the BBB (*k*_*w*_) divided by CBF and the right side is the logarithmic function of water extraction fraction ($-\text{l}\text{n}[1-{E}_{w}]$) per capillary volume (*V*_*c*_), indicating the BBB function adjusted for CBF values [36]. All the calculations to create these parametric maps were performed using our in-house scripts run with MATLAB R2021a (MathWorks, Natick, MA).

### Atlas-based analysis

To create the QSM atlas for each subject and to quantify mean magnetic susceptibility values, the multi-atlas QSM library available through the MRICloud platform (https://mricloud.org/) was used. Sixteen anatomical regions, including both sides of the caudate nucleus, putamen, globus pallidus external/internal, thalamus, pulvinar, substantia nigra, and red nucleus, were automatically segmented based on the multi-atlas label-fusion algorithm [37]. For the assessment of regional BBB function, the QSM atlas of each subject was transformed to the *k*_*w*_ and *k*_*w*_/CBF maps using the rigid transformation matrix between the magnitude image of the first echo and the proton density image (Fig. S1 in Additional file 1). Then, the mean *k*_*w*_ and *k*_*w*_/CBF values of the 16 anatomical regions were measured from each parametric map of all the subjects. These quantitative MRI parameters were also measured from subjects who were not administered sedation at all.

### Statistical analysis

The primary objective of this study was to examine whether there is a relationship between brain iron dynamics and BBB function during childhood. Considering the accumulative nature of brain iron in the deep gray-matter nuclei, we were interested in the rate of change for regional brain iron deposition over months of age (Δsusceptibility value) rather than the total amount of iron (bulk susceptibility) [11]. Therefore, first, scattergrams plotted between the susceptibility values and months of age of all the participants were reviewed in the 16 anatomical regions. Second, four types of polynomial regression models (linear, quadratic, cubic, and quartic) were fitted as a function of age. The Akaike’s Informational Criterion [38] was applied to select the best-fitted model that represented a developmental trajectory of susceptibility values in each anatomical region. Third, the best-fitted model was differentiated with respect to age. Finally, Δsusceptibility values were derived from the derivative function of the best-fitted model with substitution at each age of all the participants.

To demonstrate the extent to which the choice of assumed ATT affects the *k*_*w*_ values, a sensitivity analysis of ATT varying from 1000 ms to 1800 ms in steps of 400 ms was performed for the 16 anatomical regions in all the subjects.

For the assessment of the relationship between the rate of change for regional brain iron deposition and the water exchange rate across the BBB, scattergrams between the Δsusceptibility and *k*_*w*_/CBF values were plotted and the best-fitted regression models were chosen by the Akaike’s Informational Criterion. Then, the root-mean-square error of each best-fitted regression model was compared to that of each sigmoidal-fitting curve model. If the sigmoidal curve model was best fitted, the *k*_*w*_/CBF value of the inflection point was extracted for each anatomical region, and regional differences in these inflection points were visualized. All the statistical analyses were conducted in Python 3.9.12 and Stata 16.0 (StataCorp, College Station, TX).

## Results

### Participant characteristics

**Fig. 1:**
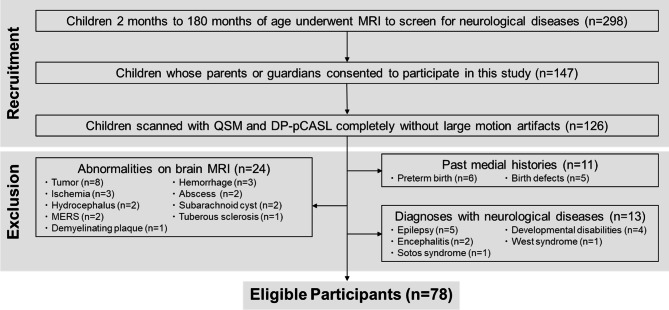
Flowchart of participant recruitment and exclusion. DP-pCASL = diffusion-prepared pseudo-continuous arterial spin labeling, MERS = mild encephalitis/encephalopathy with reversible splenial lesion, QSM = quantitative susceptibility mapping

A total of 298 children underwent brain MRI to screen for neurological diseases during the recruitment period. Among them, children whose parents or guardians consented to participate in this study (*n* = 147) and scanned with QSM and DP-pCASL completely without large motion artifacts (*n* = 126) were included (Fig. 1). Then, children with histories of preterm birth (*n* = 6) or birth defects (*n* = 5), or visible abnormalities on brain MRI, such as tumor (*n* = 8), hemorrhage (*n* = 3), ischemia (*n* = 3), abscess (*n* = 2), hydrocephalus (*n* = 2), subarachnoid cyst (*n* = 2), mild encephalitis/encephalopathy with reversible splenial lesion (*n* = 2), tuberous sclerosis (*n* = 1), and demyelinated plaque (*n* = 1), were excluded. In addition, children who were diagnosed with neurological diseases during follow-up examinations, such as epilepsy (*n* = 5), developmental disabilities (*n* = 4), encephalitis (*n* = 2), West syndrome (*n* = 1), and Sotos syndrome (*n* = 1), were also excluded. Thus, a total of 78 children (mean age, 73.8 months ± 61.5 [SD]; 43 boys) were eligible for the following evaluations. Demographics and clinically suspected brain diseases are provided in Table 1.

**Table 1:** Demographic Characteristics of Participants

Characteristics	Eligible Participants
Total	78
Months of age	73.8 ± 61.5 (2–180)
2–24 months	27
25–99 months	24
100–180 months	27
Sex	
Boy	43
Girl	35
Suspected brain diseases	
Tumor	28
Stroke	21
Epilepsy	18
Malformation	5
Encephalitis	4
Metabolic disease	2

Note: Data are numbers of participants, or mean ± SD with range in parenthesis

### Representative images

**Fig. 2:**
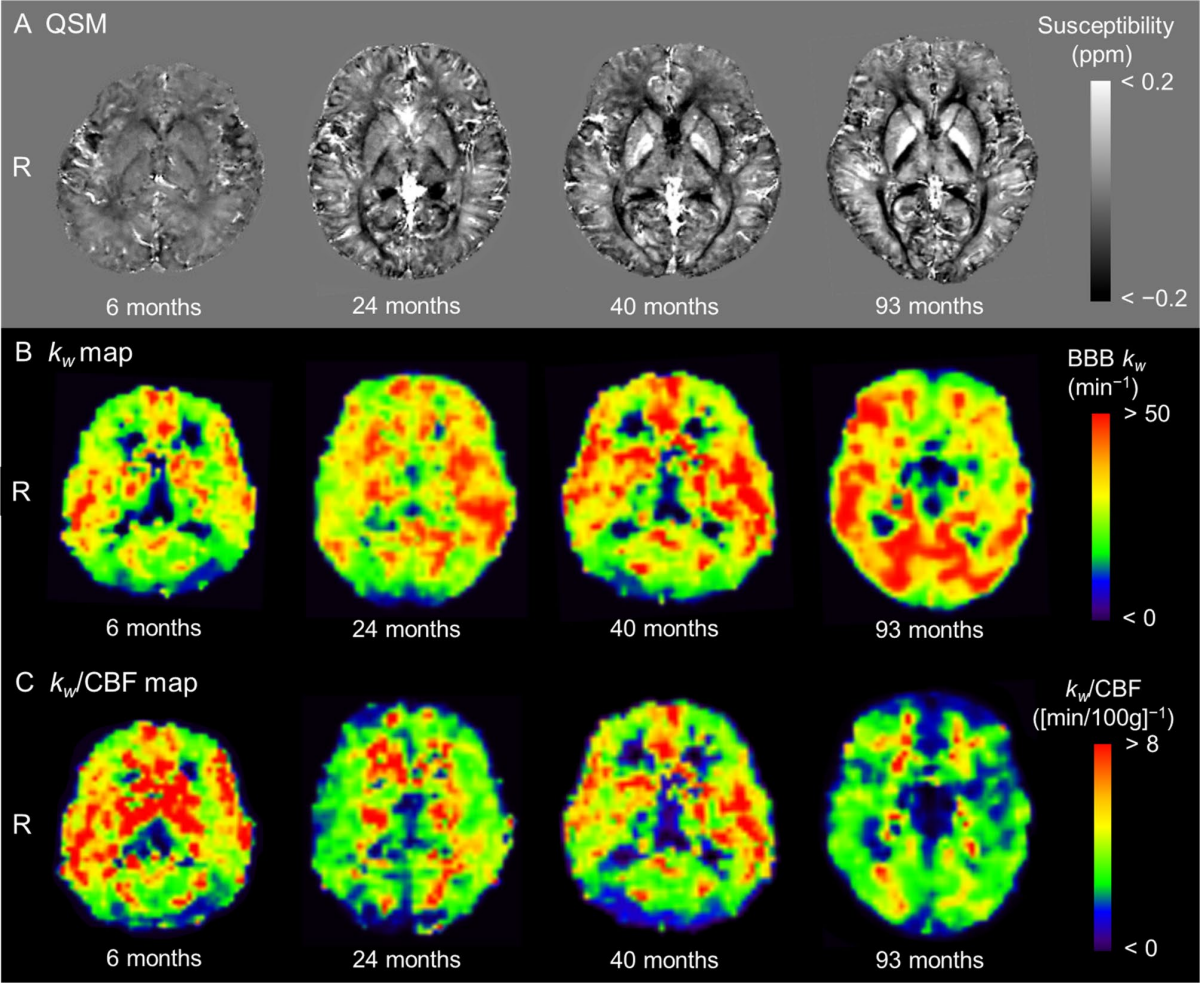
Representative images. QSM (A) and corresponding *k*_*w*_ (B) and *k*_*w*_/CBF (C) maps obtained from eligible participants— a 6-month-old boy, 24-month-old girl, 40-month-old boy, and 93-month-old boy are displayed. In QSM, the interfaces between the deep gray-matter nuclei and surrounding white matter were only slightly visible at 6 months of age. With increasing age, these boundaries were gradually distinguishable, with the highest susceptibility value in the globus pallidus. In contrast, the differences in the *k*_*w*_ and *k*_*w*_/CBF maps were indiscernible except that the *k*_*w*_/CBF map at 6 months broadly showed high *k*_*w*_/CBF values, especially in the basal ganglia. BBB = blood-brain barrier, CBF = cerebral blood flow, QSM = quantitative susceptibility, R = right

Figure 2 displays representative images of the QSM (Fig. 2A) and corresponding *k*_*w*_ (Fig. 2B) and *k*_*w*_/CBF (Fig. 2C) maps from eligible participants at 6, 24, 40, and 93 months. At 6 months of age, the interfaces between the deep gray-matter nuclei and surrounding white matter were only slightly visible. With increasing age, these boundaries were gradually distinguishable, with the highest susceptibility value in the globus pallidus. In contrast, there were indiscernible differences in the *k*_*w*_ and *k*_*w*_/CBF maps except that the *k*_*w*_/CBF map at 6 months broadly showed high *k*_*w*_/CBF values, especially in the basal ganglia.

### Atlas-based analyses

**Fig. 3:**
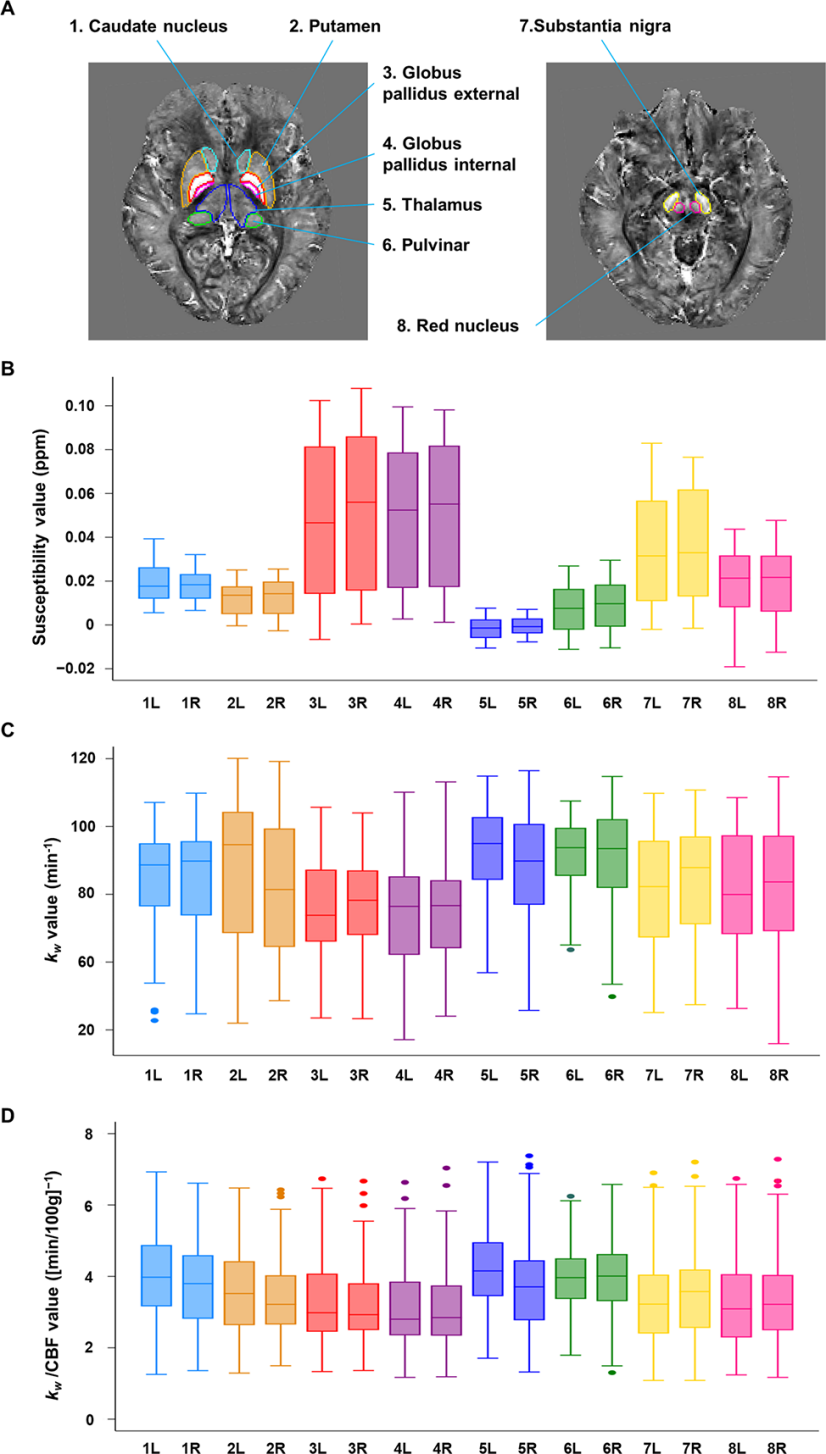
Atlas-based analysis. The selected anatomical regions in the deep gray-matter nuclei are indicated on the QSM space (A). The mean susceptibility (B), *k*_*w*_ (C), and *k*_*w*_/CBF values (D) of the 16 anatomical regions were measured from each parametric map. Each number on the horizontal axis corresponds to the number on the QSM atlas (A). CBF = cerebral blood flow, L = left, QSM = quantitative susceptibility mapping, R = right

The QSM atlas created by the multi-atlas label-fusion method successfully segmented both sides of the deep gray-matter nuclei (Fig. 3A). The mean susceptibility (Fig. 3B), *k*_*w*_ (Fig. 3C), and *k*_*w*_/CBF values (Fig. 3D) of the 16 anatomical regions were measured from each parametric map. In accordance with these visualized findings, the susceptibility value of the globus pallidus was the highest among the deep gray-matter nuclei and showed the widest range over the months of age. Meanwhile, there were no distinct differences in the *k*_*w*_ and *k*_*w*_/CBF values among the anatomical regions (Table S1 in Additional file 2). In the sedation-free subjects (*n* = 59; mean age, 86.2 months ± 43.1 [SD]; 32 boys), the mean susceptibility values were slightly increased, whereas there were no distinct differences in the mean *k*_*w*_ and *k*_*w*_/CBF values, compared to these values in all the subjects (Table S2 in Additional file 2). The sensitivity analysis showed that ± 30% changes in ATT affected ± 30% changes in *k*_*w*_ values in the 16 anatomical regions (Table S3 in Additional file 2).

### Developmental trajectories of susceptibility value

**Fig. 4:**
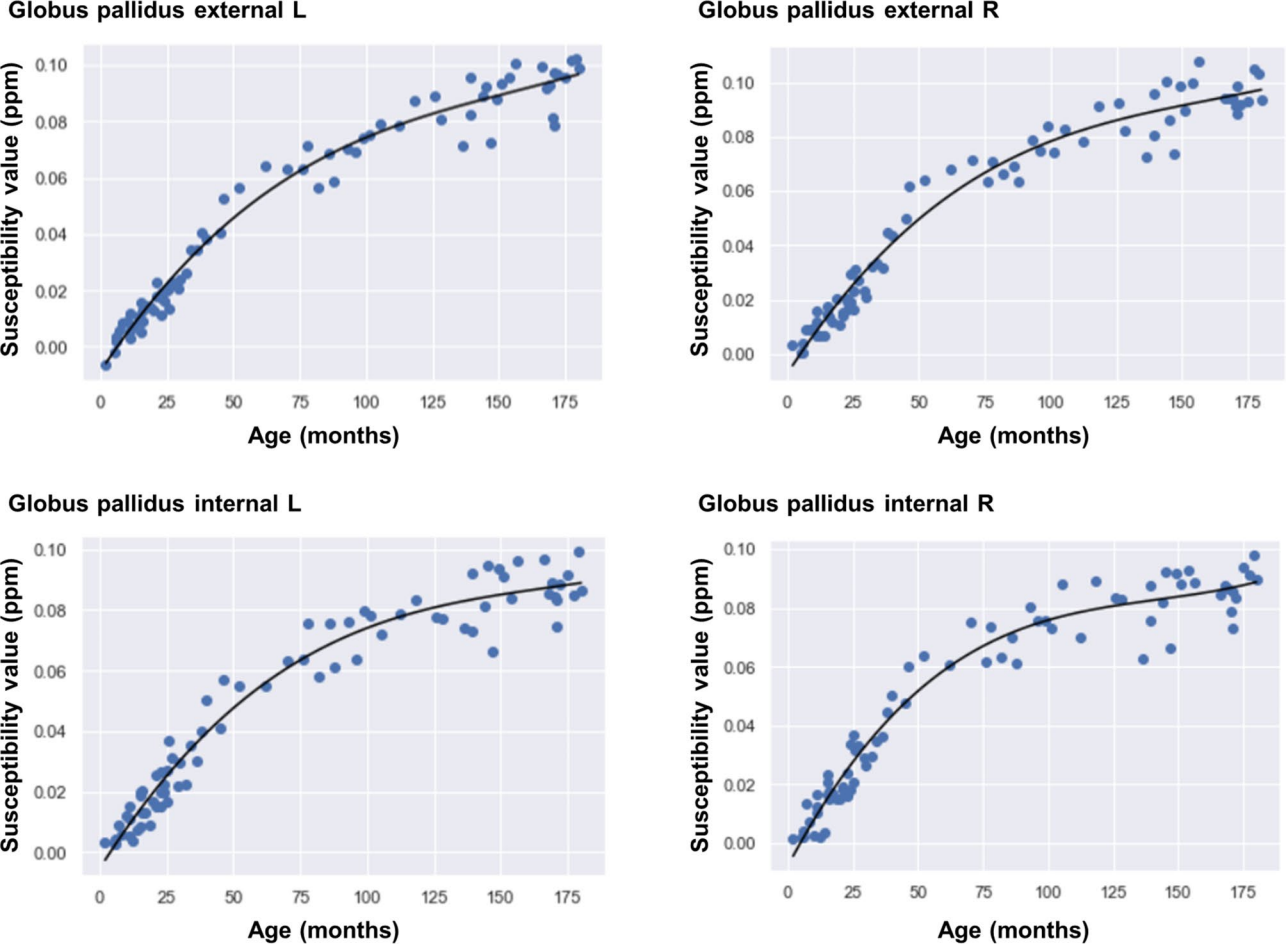
Scattergrams plotting the susceptibility value and months of age in the globus pallidus and the best-fitted regression models. L = left, R = right

Scattergrams of the 16 anatomical regions between the susceptibility values and months of age are shown in Fig. S2 (Additional file 1). Rapid magnetic susceptibility progression in each anatomical region was observed during the first two years. The best-fitted models as functions of age in the globus pallidus are displayed in Fig. 4 and those of the 16 anatomical regions are summarized in Table S4 (Additional file 2). Some models were cubic equations and others were quadratic equations. These derivative functions are also summarized in Table S4 (Additional file 2). With the substitution at each age of all the participants, the regional Δsusceptibility value was calculated in each group of participants for 2–24 months, 25–99 months, and 100–180 months (Table 2). With increasing age, the Δsusceptibility values were decreased in all the anatomical regions. The globus pallidus of the 2–24 months group showed the highest Δsusceptibility value of 1.26 ± 0.18 (× 10^− 3^ ppm/month), whereas most regions of the 100–180 months group, except the globus pallidus and substantia nigra, had low Δsusceptibility values close to zero, indicating that these susceptibility values had reached the plateaus.

**Table 2:** ΔSusceptibility Value of Anatomical Region

	ΔSusceptibility Value (× 10^− 3^ ppm/month)		
Anatomical Region	2–24 (months)	25–99 (months)	100–180 (months)
Caudate nucleus L	0.23 ± 0.07	0.17 ± 0.04	0.04 ± 0.02
Caudate nucleus R	0.21 ± 0.05	0.15 ± 0.04	0.03 ± 0.02
Putamen L	0.31 ± 0.08	0.17 ± 0.05	0.04 ± 0.03
Putamen R	0.39 ± 0.11	0.19 ± 0.07	0.07 ± 0.04
Globus pallidus external L	1.22 ± 0.19	0.77 ± 0.25	0.27 ± 0.08
Globus pallidus external R	1.28 ± 0.20	0.79 ± 0.27	0.22 ± 0.07
Globus pallidus internal L	1.19 ± 0.18	0.74 ± 0.26	0.16 ± 0.06
Globus pallidus internal R	1.38 ± 0.23	0.76 ± 0.28	0.17 ± 0.06
Thalamus L	0.15 ± 0.04	0.10 ± 0.03	0.02 ± 0.01
Thalamus R	0.16 ± 0.05	0.08 ± 0.04	0.03 ± 0.02
Pulvinar L	0.27 ± 0.07	0.21 ± 0.05	0.05 ± 0.04
Pulvinar R	0.29 ± 0.09	0.22 ± 0.06	0.04 ± 0.02
Substantia nigra L	0.73 ± 0.15	0.50 ± 0.10	0.26 ± 0.07
Substantia nigra R	0.63 ± 0.13	0.49 ± 0.09	0.23 ± 0.06
Red nucleus L	0.58 ± 0.11	0.34 ± 0.07	0.14 ± 0.05
Red nucleus R	0.60 ± 0.12	0.34 ± 0.08	0.10 ± 0.03

Note: Data are means ± standard deviation

Abbreviation: L = left, R = right

### Developmental trajectories of BBB function parameters

Scattergrams of the 16 anatomical regions plotting the BBB function parameters (*k*_*w*_ and *k*_*w*_/CBF values) and CBF against months of age are shown in Fig. S3, Fig. S4, and Fig. S5, respectively (Additional file 1). The *k*_*w*_ values had broad ranges of 35 to 120 min^− 1^ and showed no direct associations with age. In line with the previous studies using an arterial spin labeling technique [39], age-related evolution of CBF values were observed along 2–180 months of age, with broad ranges of 10 to 55 mL/100 g/min. In contrast, the *k*_*w*_/CBF values decreased rapidly during the first few years and were gradually decreased thereafter.

### Relationship between Δsusceptibility and *k*_*w*_ /CBF values

**Fig. 5:**
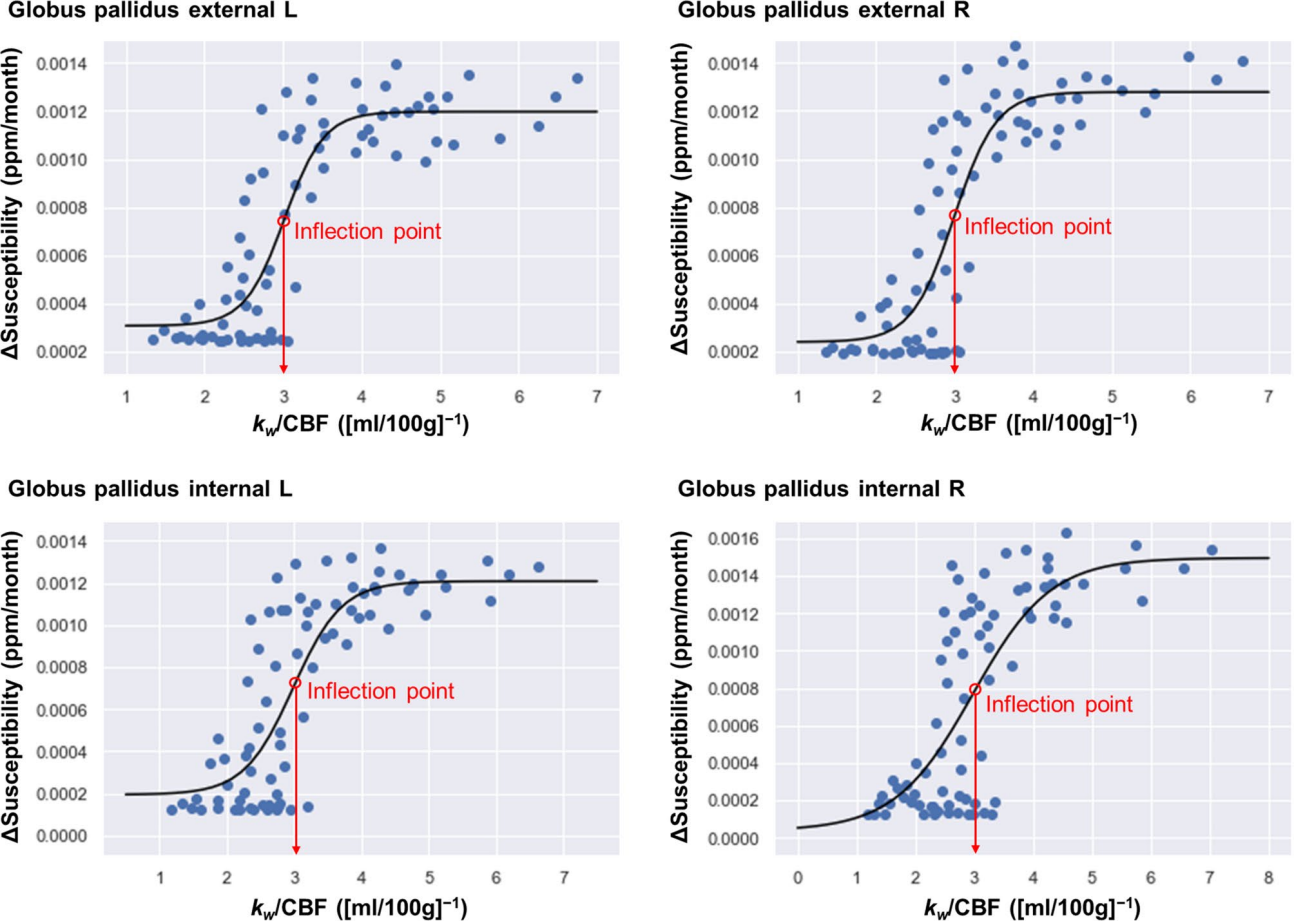
Scattergrams plotting the Δsusceptibility and *k*_*w*_/CBF values in the globus pallidus and sigmoidal curve-fitting models. Red circles and arrows indicate *k*_*w*_/CBF values of inflection points in these sigmoidal curves. CBF = cerebral blood flow, L = left, R = right

**Fig. 6:**
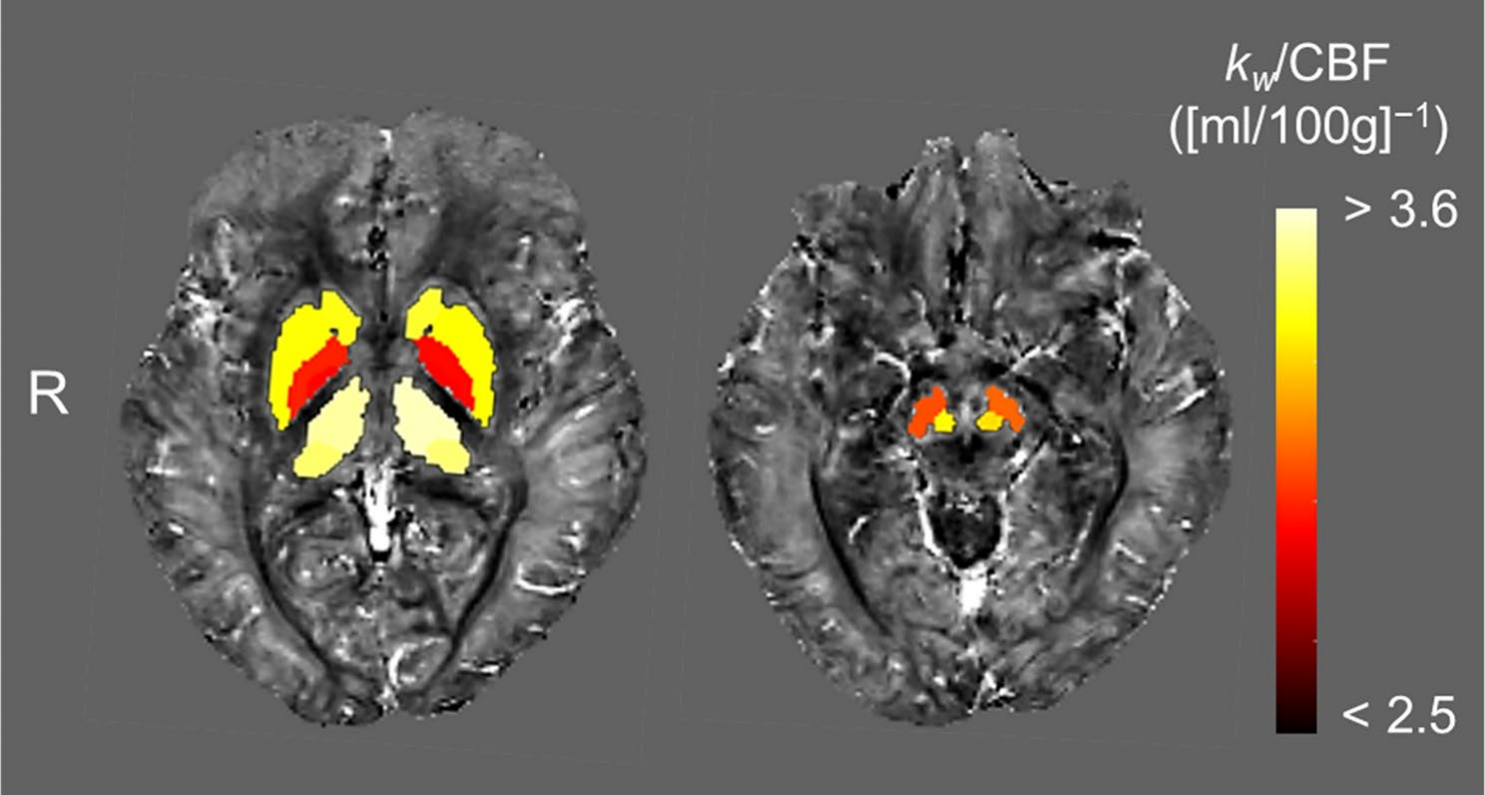
Inflection points overlaid on QSM. The *k*_*w*_/CBF value of the inflection point in each anatomical region was overlaid on QSM. This map visualized regional differences in these inflection points, consistent with the regional heterogeneity of brain iron accumulation. CBF = cerebral blood flow, R = right

Scattergrams between the Δsusceptibility and *k*_*w*_ /CBF values are shown in Fig. 5 (globus pallidus) and Fig. S6 (other regions, Additional file 1). In all the regions, the root-mean-square error of the sigmoidal-fitting curve model was lower than that of the best-fitted regression model chosen by the Akaike’s Informational Criterion (Table S5 and Table S6 in Additional file 2). The *k*_*w*_/CBF value of the inflection point was extracted from the sigmoidal curve in each anatomical region and overlaid on QSM (Fig. 6). This map visualized regional differences in these inflection points, consistent with the regional heterogeneity of brain iron accumulation. The globus pallidus had the lowest inflection point of 2.96–3.03 (mL/100 g)^−1^ and the substantia nigra had the second lowest of 3.12–3.15, whereas the thalamus had the highest of 3.64–3.67 (Table S7 in Additional file 2). These results demonstrated that the more prone each anatomical region was to brain iron accumulation, the lower the *k*_*w*_/CBF value of the inflection point in the sigmoidal curve.

## Discussion

There is a lack of human studies to examine the relationship between brain iron deposition and BBB function during childhood. The present study directly compared both physiological systems along the developmental trajectory using QSM and DP-pCASL in pediatric brains. We quantified the rate of change for brain iron deposition as the Δsusceptibility value and the water exchange rate across the BBB as the *k*_*w*_/CBF value and found that their scattergrams in the deep gray-matter nuclei were fitted to sigmoidal curve models. The *k*_*w*_/CBF value of the inflection point extracted from the sigmoidal curve differed among each deep gray-matter nucleus, consistent with the regional heterogeneity of brain iron accumulation.

Regional heterogeneities of the interaction between brain iron dynamics and BBB function supported a concept of organized BBB functionality during normal brain development. For many years, it was believed that iron entered the brain tissue intensively during the fetal and neonatal periods before the BBB matured [6]. However, it has become apparent that the tight junctions between the endothelial cells of the brain capillary wall are present as early as eight weeks gestation in human fetuses and restrict the passage of low-weight molecules, including ferrous and ferric ions [40]. Rather than the widespread belief that the BBB is “immature” during early development, there has been physiological evidence that iron transport across the BBB is functionally activated in the developing brain [2, 41], in line with the results of the present study. In contrast to the iron transport system into the brain, there has been little evidence regarding the clearance system of iron through the BBB. Some studies have suggested that iron could be transported from the brain to the cerebrospinal fluid, contributing to its clearance [42–44].

To the best of our knowledge, this study is the first to present quantitative values of the water exchange rate across the BBB for pediatric brains. Our results of *k*_*w*_ values varied widely over months of age and were influenced by age-related changes of CBF values [39]. CBF is closely associated with the BBB function parameters because *k*_*w*_ values are calculated from the ratio between intravascular and extravascular labeled protons [17, 45]. Therefore, we also output the *k*_*w*_/CBF index to assess the BBB function adjusted for CBF values. The *k*_*w*_/CBF values showed a characteristic relationship with age and were associated with the Δsusceptibility values as sigmoidal curve-fitting models. A number of biological studies used sigmoidal fitting to analyze dose-response relationships [46], the competition of a ligand for receptor binding [47], or the voltage-dependent activation of ion channels [48]. Interestingly, the scattergram between serum ferritin and the amount of storage iron was also fitted to the sigmoidal curve [49].

The reason that the scattergrams between the Δsusceptibility and *k*_*w*_/CBF values were best fitted to sigmoidal curve models remains unclear. Brain iron concentration is dependent on brain iron uptake and clearance from the brain. One possibility is a direct link between the *k*_*w*_/CBF index and brain iron uptake via iron transporters on BBB endothelial cells [1]. However, the *k*_*w*_/CBF index is thought to reflect the water exchange rate across the BBB through perivascular AQP4 channels [21], which would be unable to regulate the transport of ferrous and ferric ions directly [50]. Another possibility is a confounding effect of an iron transporter on the association between the Δsusceptibility and *k*_*w*_/CBF values. The upregulated transport of iron into the brain tissue due to a particular need for iron during normal brain growth [2, 41] would have influences on both Δsusceptibility and *k*_*w*_/CBF values, considering the close relationship between iron and AQP4 channels [7–10]. Conversely, the increased brain iron in aging and neurodegenerative diseases was correlated with decreased BBB water exchange rate as the *k*_*w*_ index [51, 52], which could be the result of dysfunctions of the perivascular AQP4 channels [53]. The distribution and expression of AQP4 channels could have an important role in the glymphatic brain-waste clearance pathway [54]. Taken together, the interaction between brain iron dynamics and BBB function in the developing brain seems to be different from that in the elderly [21].

Our study had several limitations. First, the MRI scans were obtained based on clinical indications, and QSM and DP-pCASL sequences were add-on protocols. This included the following two disadvantages: (1) the recruitment inevitably had a selection bias and further follow-up examinations beyond December 2022 may have revealed additional participants with neurological diseases. Therefore, we could not establish normative quantitative MRI measures such as *k*_*w*_ and *k*_*w*_/CBF values across 2–180 months of age. Nonetheless, the results of the susceptibility and CBF values in this study were in agreement with those of previous studies [13–16, 39]. (2) We could not evaluate the test-retest reliability of *k*_*w*_ values derived from the proposed DP-pCASL technique although the good reproducibility of *k*_*w*_ measurements has been reported in a previous paper [17]. Second, the cross-sectional design could not prove a causal association between BBB function and brain iron concentration [55]. Longitudinal studies should be undertaken to explore this. Third, sedation procedures for some participants could have altered brain perfusion and BBB function [56]. Fourth, we could not calculate ATT for each subject because the scan protocol for DP-pCASL included only one PLD of 1800 ms due to the limited scan time in clinical settings. The fixed ATT of 1800 ms for the quantification of *k*_*w*_ values might be inappropriate for some younger children [57].

In conclusion, the combined quantitative MRI study of QSM and DP-pCASL for pediatric brains demonstrated the relationship between brain iron dynamics and BBB function during childhood, highlighting the potential of these imaging techniques to assess both physiological systems during postnatal brain development.

### Electronic supplementary material

Below is the link to the electronic supplementary material.

Additional file 1

Additional file 2

## Data Availability

Data generated or analyzed during the study are available from the corresponding author upon reasonable request.
