# Supplementary material for: Relationship between brain iron dynamics and blood-brain barrier function during childhood: a quantitative magnetic resonance imaging study
Source: Fluids Barriers CNS. 2023 Aug 17;20:60. doi: 10.1186/s12987-023-00464-x (PMC10433620; doi:10.1186/s12987-023-00464-x)
Supplement: Supplementary file 1 — Additional file 1 [file 12987_2023_464_MOESM1_ESM.docx]

**Supplementary figures:**

**Fig. S1: Registration process of QSM atlas to *k_w_* and *k_w_*/CBF maps**

**
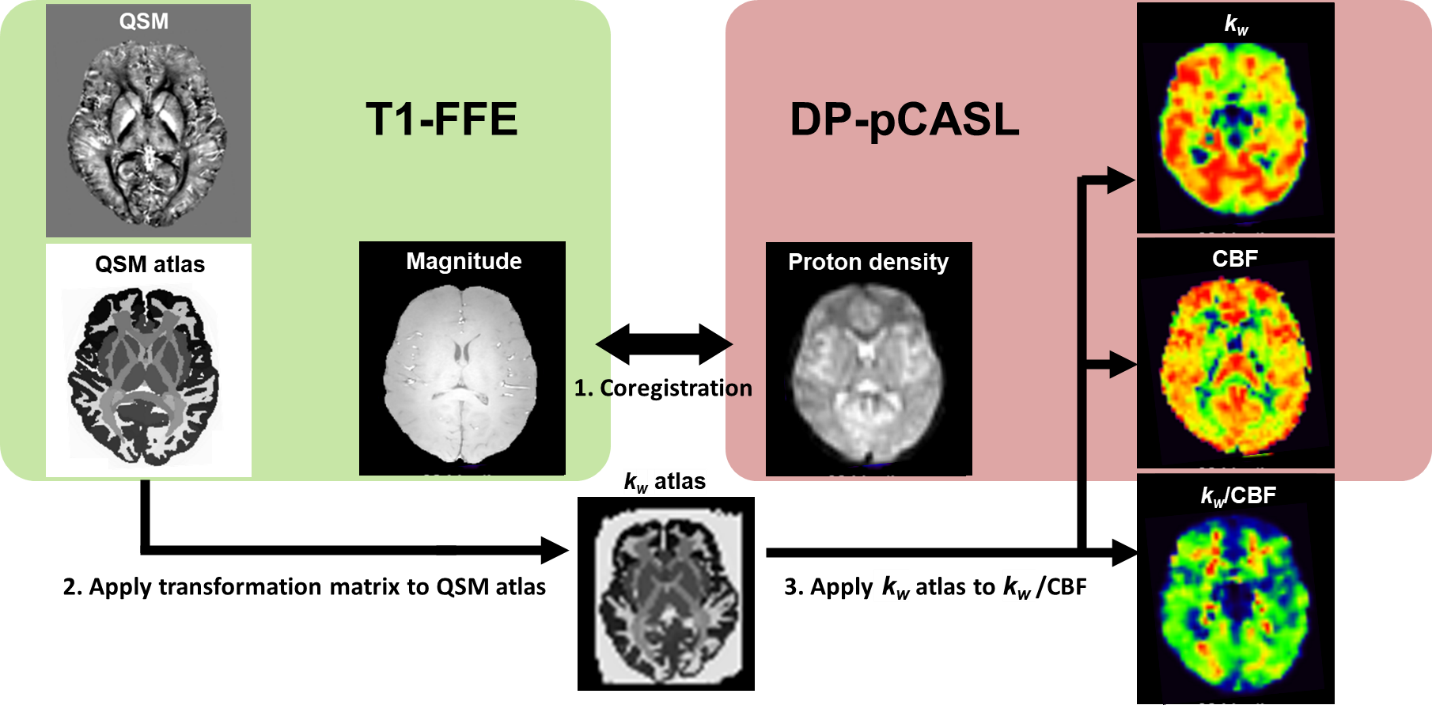
**

First, the coregistration between the first echo magnitude image extracted by the T1-FFE sequence and the proton density image created by the DP-pCASL sequence were performed to obtain the transformation matrix. Second, the transformation matrix was applied to the QSM atlas to warp into the *k_w_* space. This atlas could be useful in the parametric maps derived by the DP-pCASL sequence because of the same geometries. These processes were conducted using the DiffeoMap software (www.MRIstudio.org). CBF = cerebral blood flow, DP-pCASL = diffusion-prepared pseudo-continuous arterial spin labeling, FFE = fast field echo, QSM = quantitative susceptibility mapping.

**Fig. S2: Scattergrams plotting susceptibility values and months of age**

**
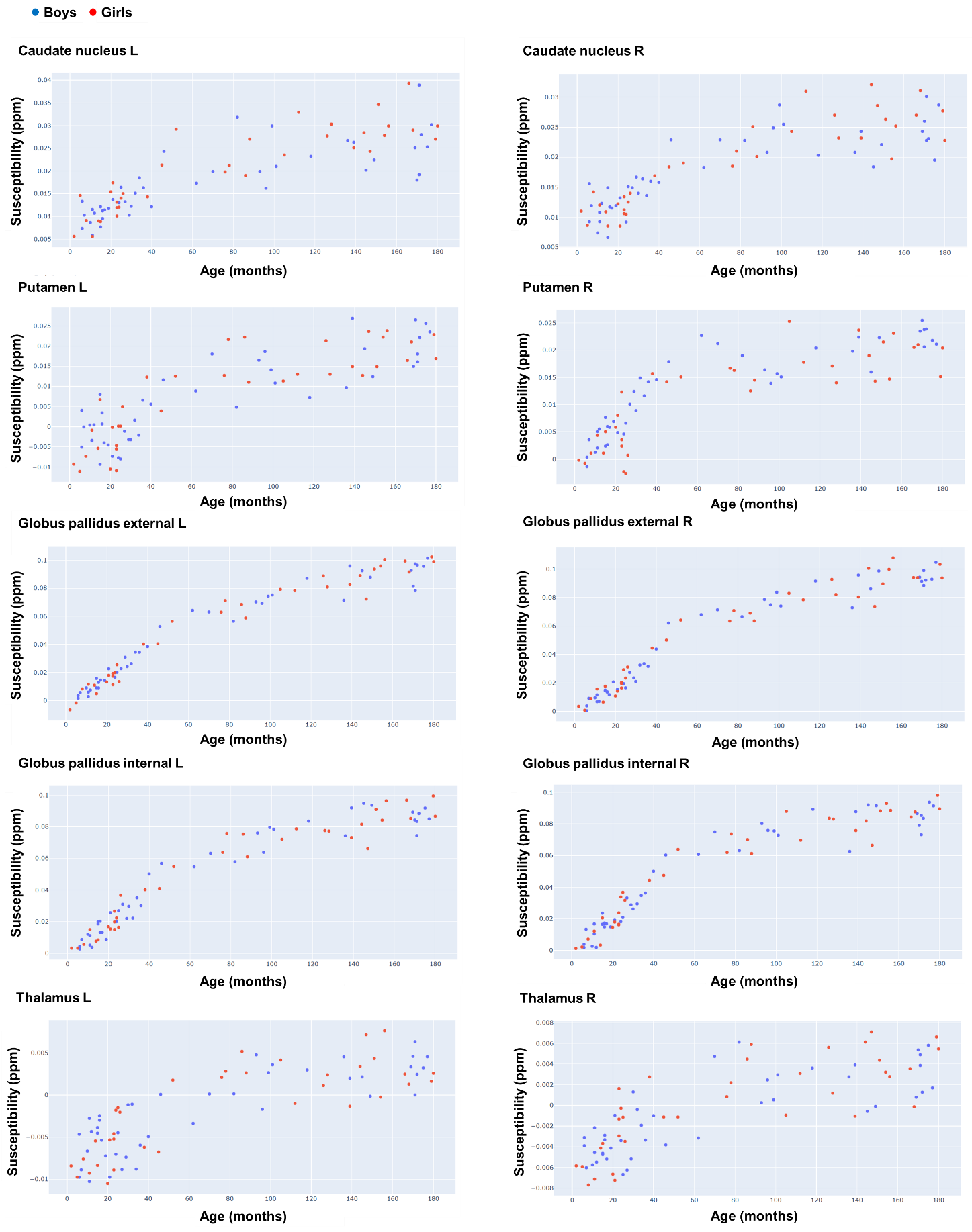
**

**
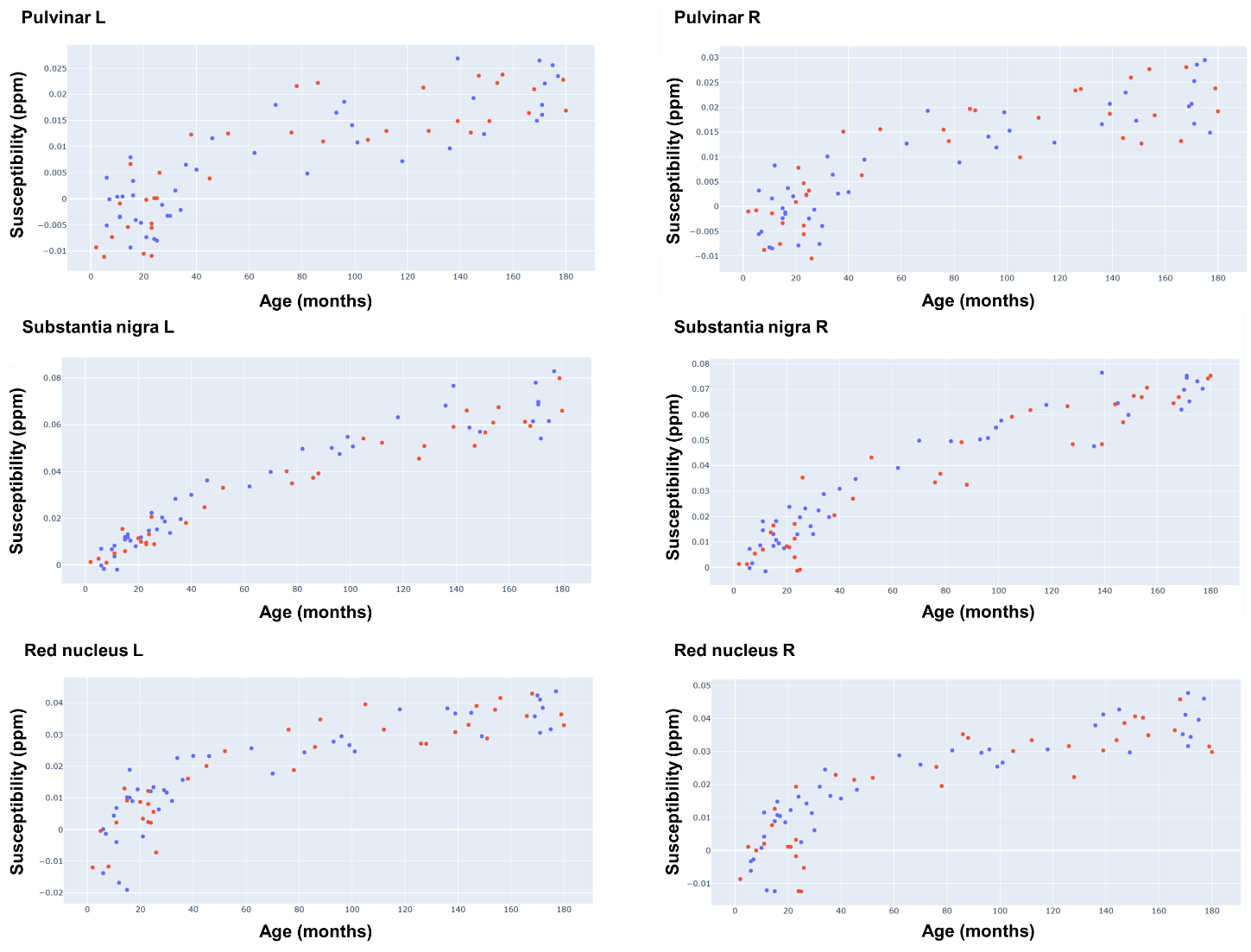
**

L = left, R = right.

**Fig. S3: Scattergrams plotting the *k_w_* values and months of age** **
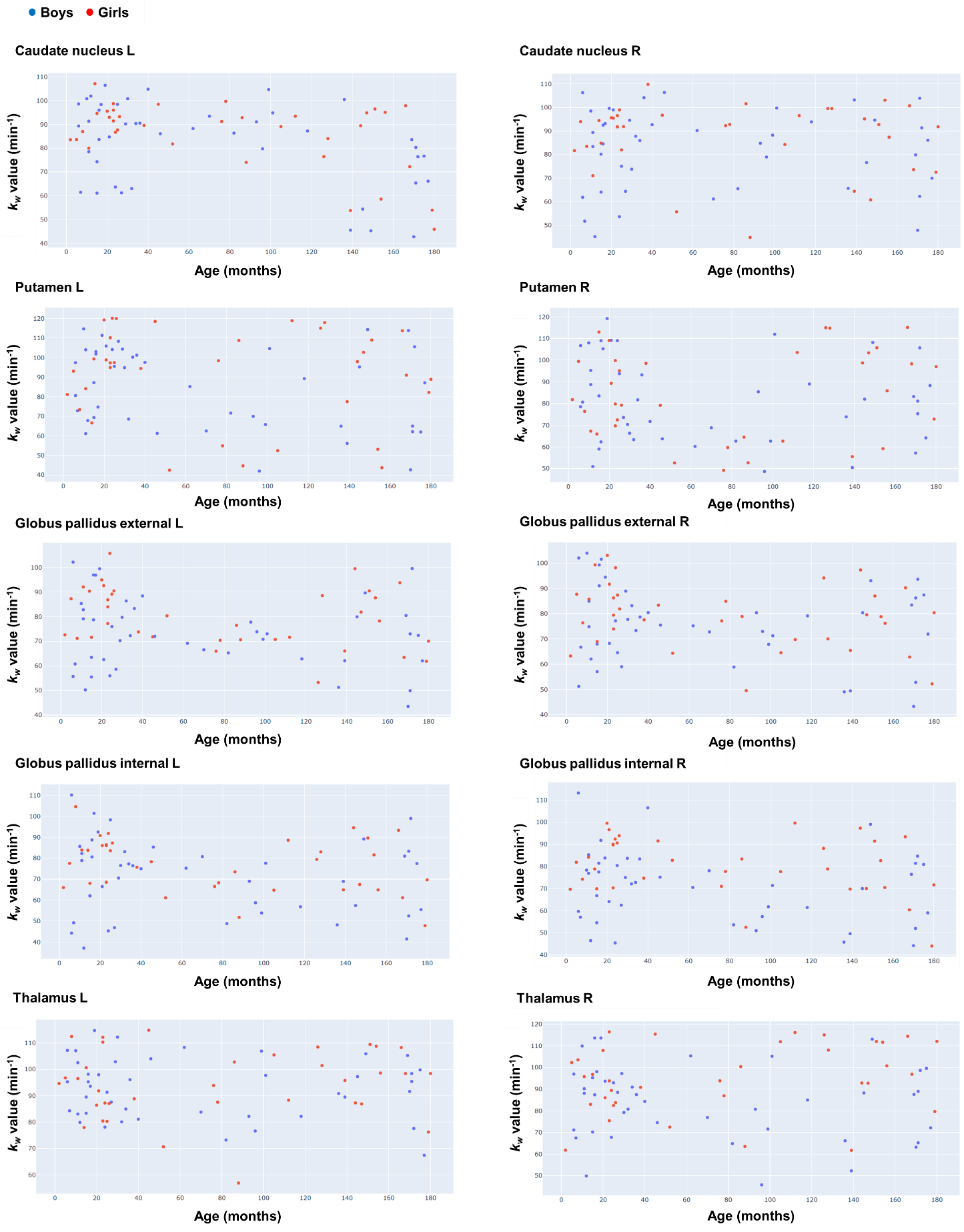

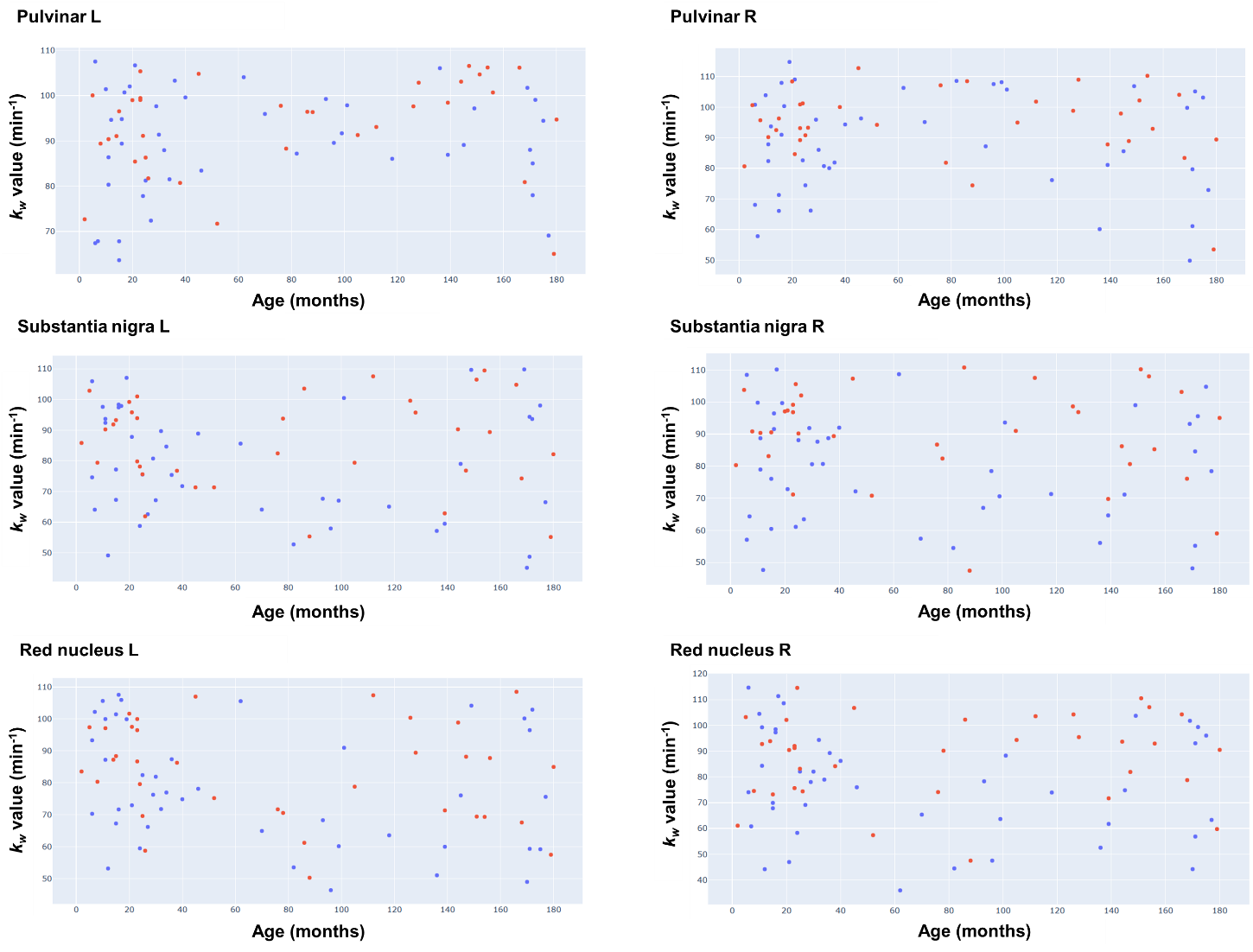
**L = left, R = right.

**Fig. S4: Scattergrams plotting the *k_w_*/CBF values and months of age
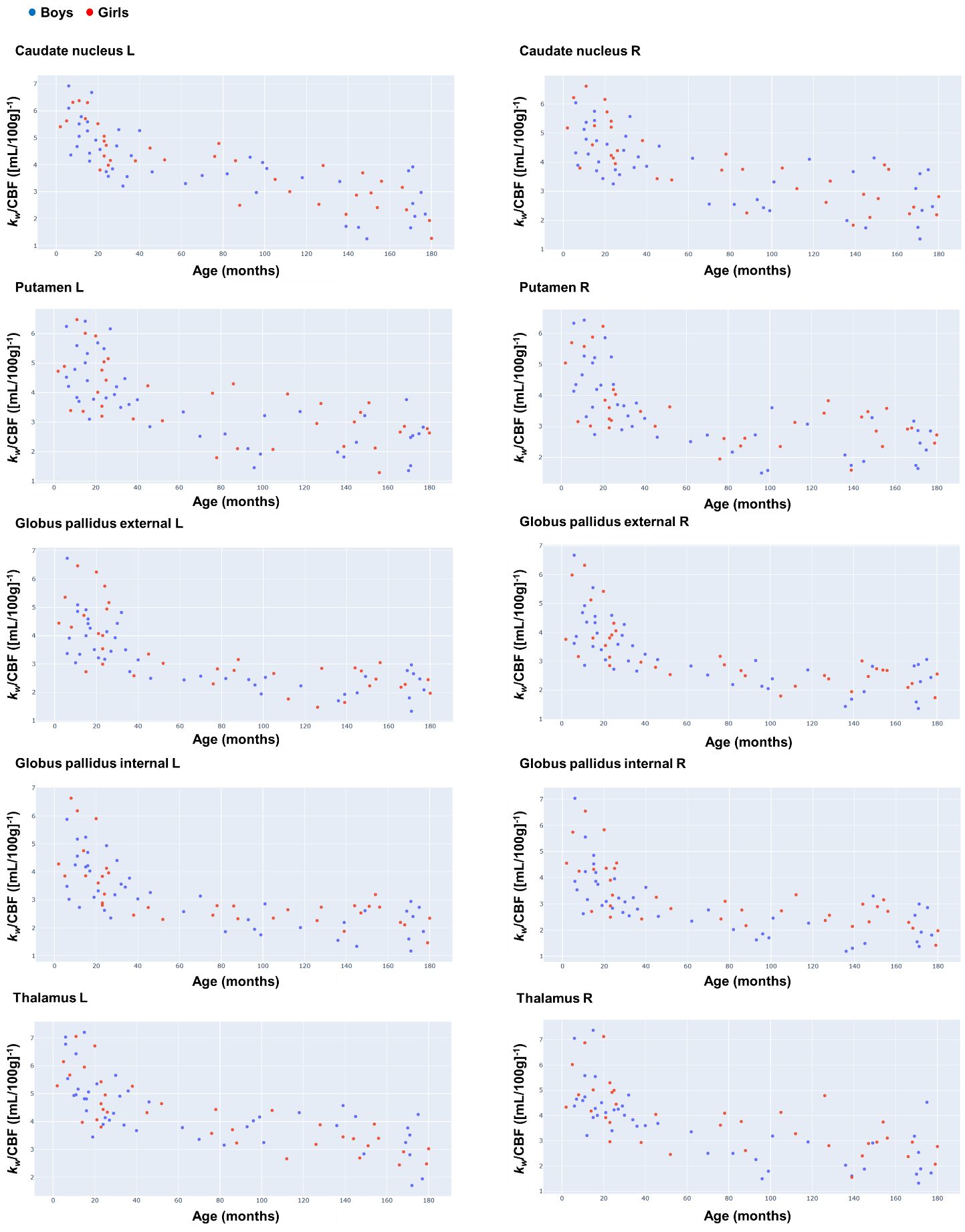
**

**
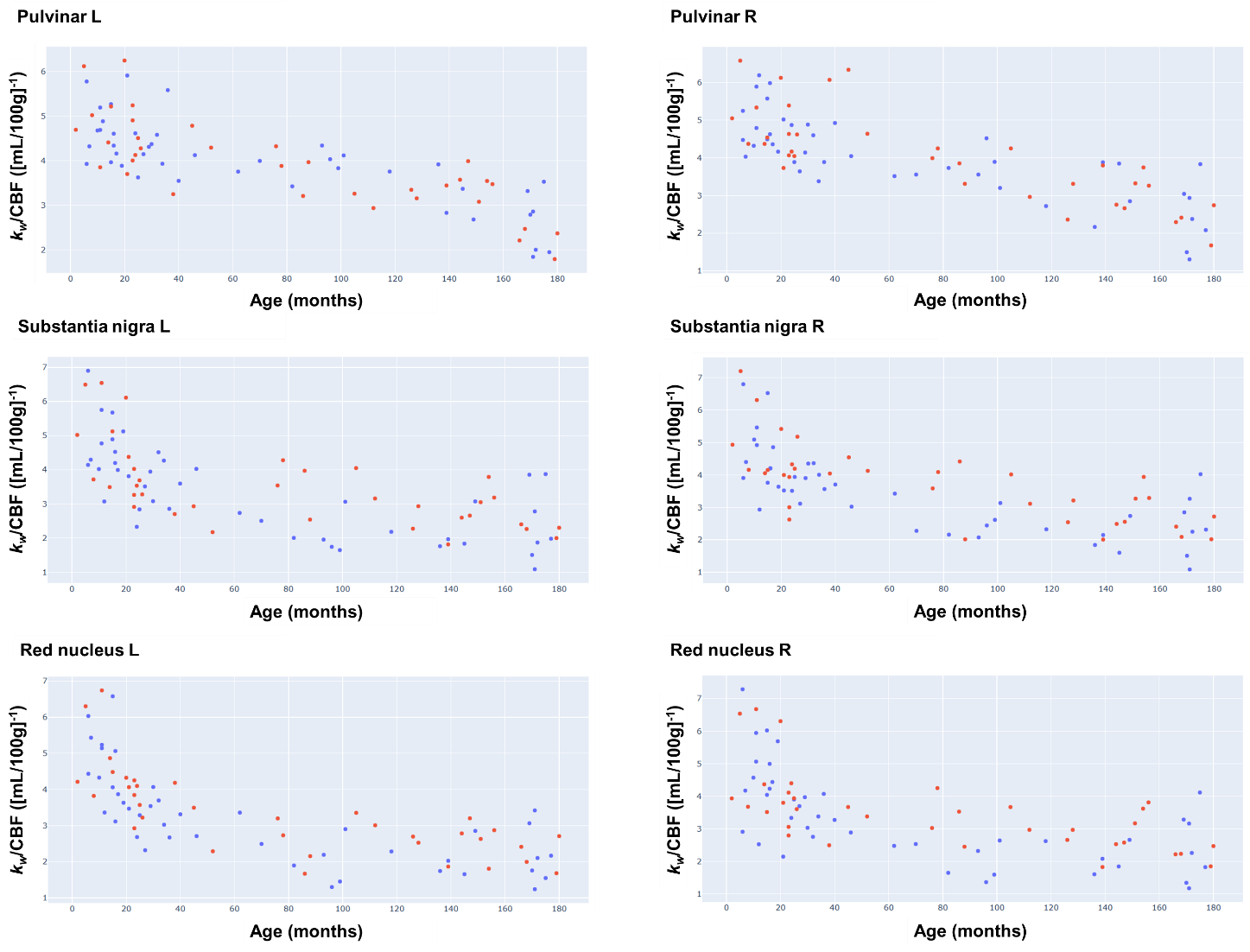
**

CBF = cerebral blood flow, L = left, R = right.

**Fig. S5: Scattergrams plotting the CBF values and months of age**


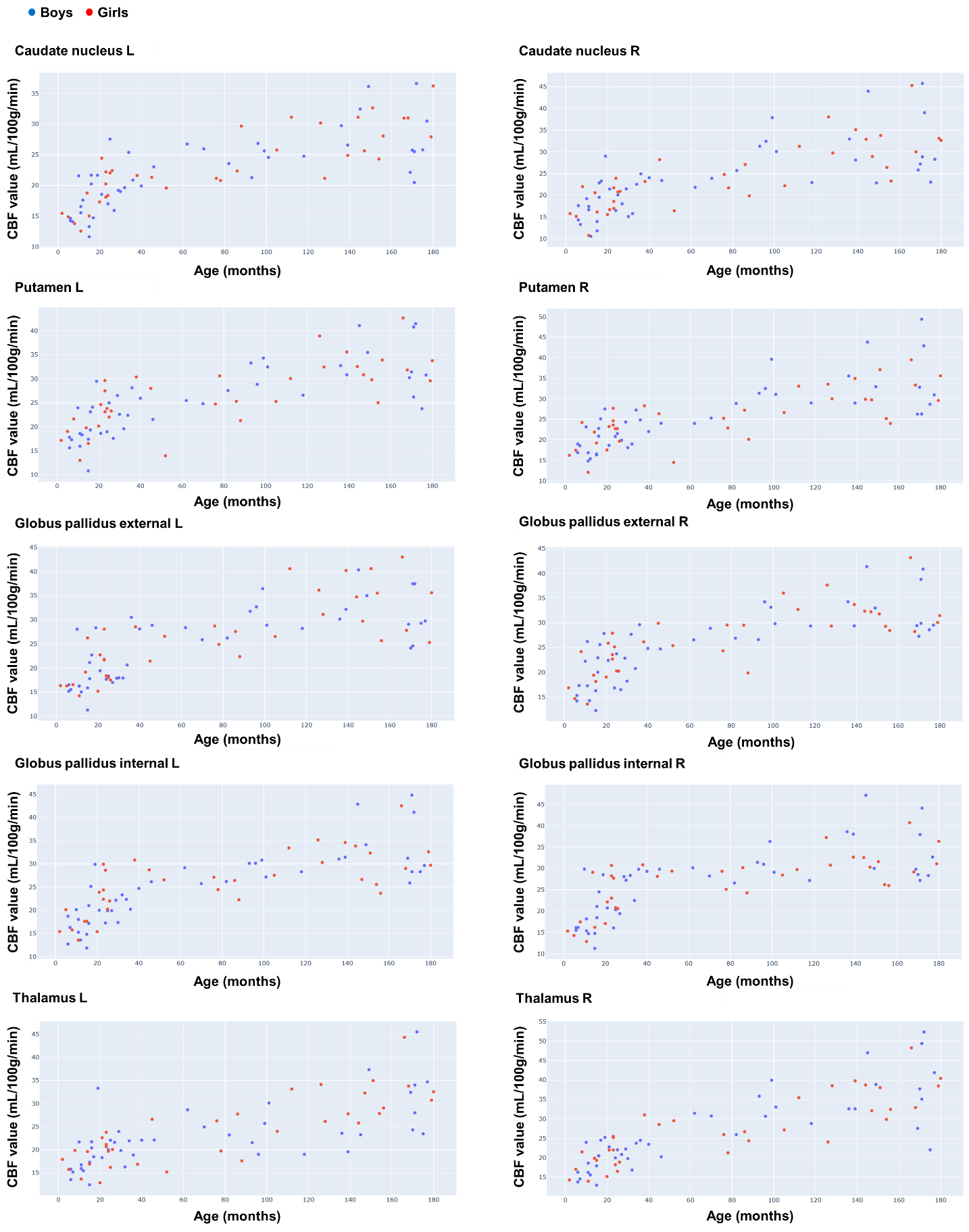


**
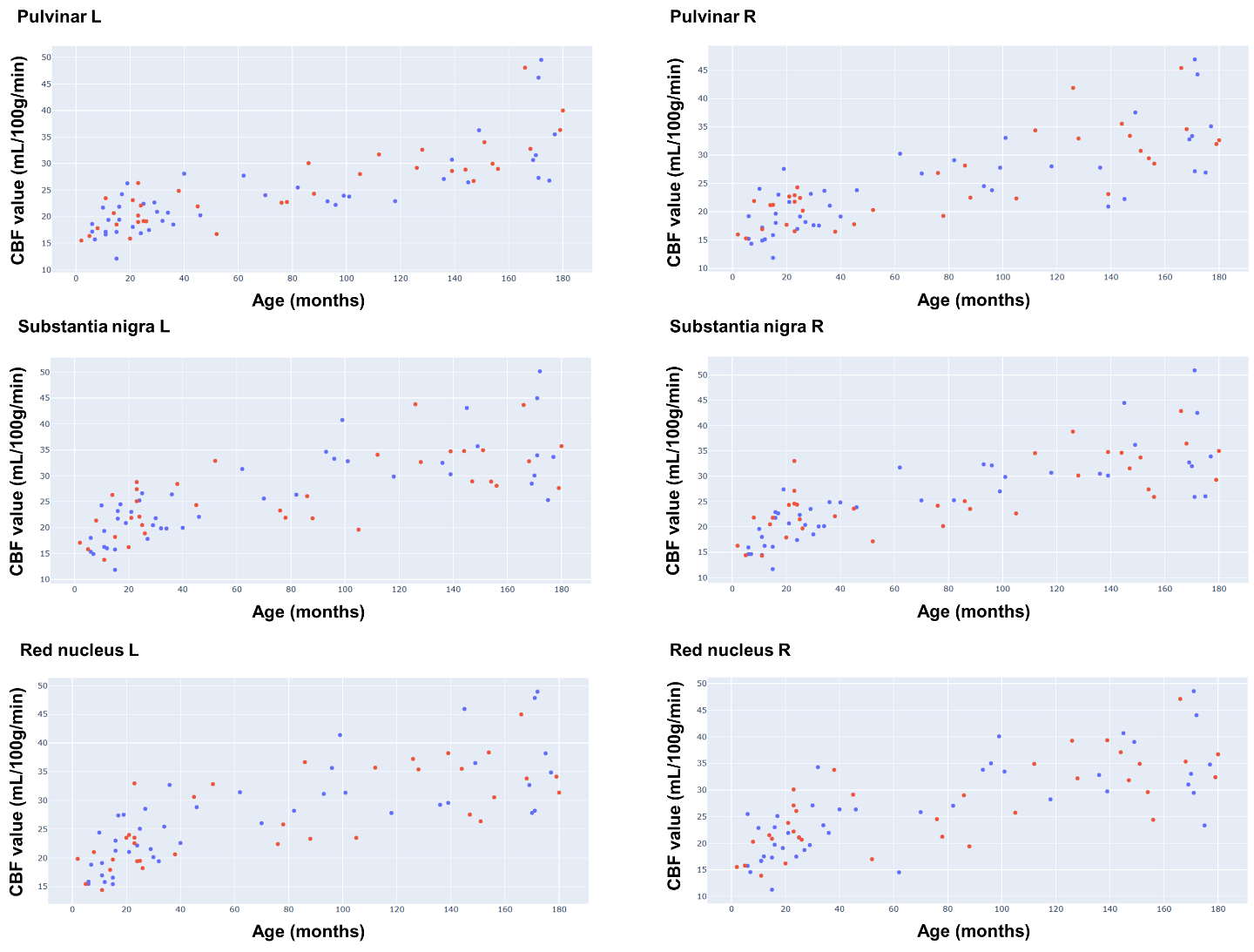
** CBF = cerebral blood flow, L = left, R = right.

**Fig. S6: Scattergrams plotting the Δsusceptibility and *k_w_*/CBF values**
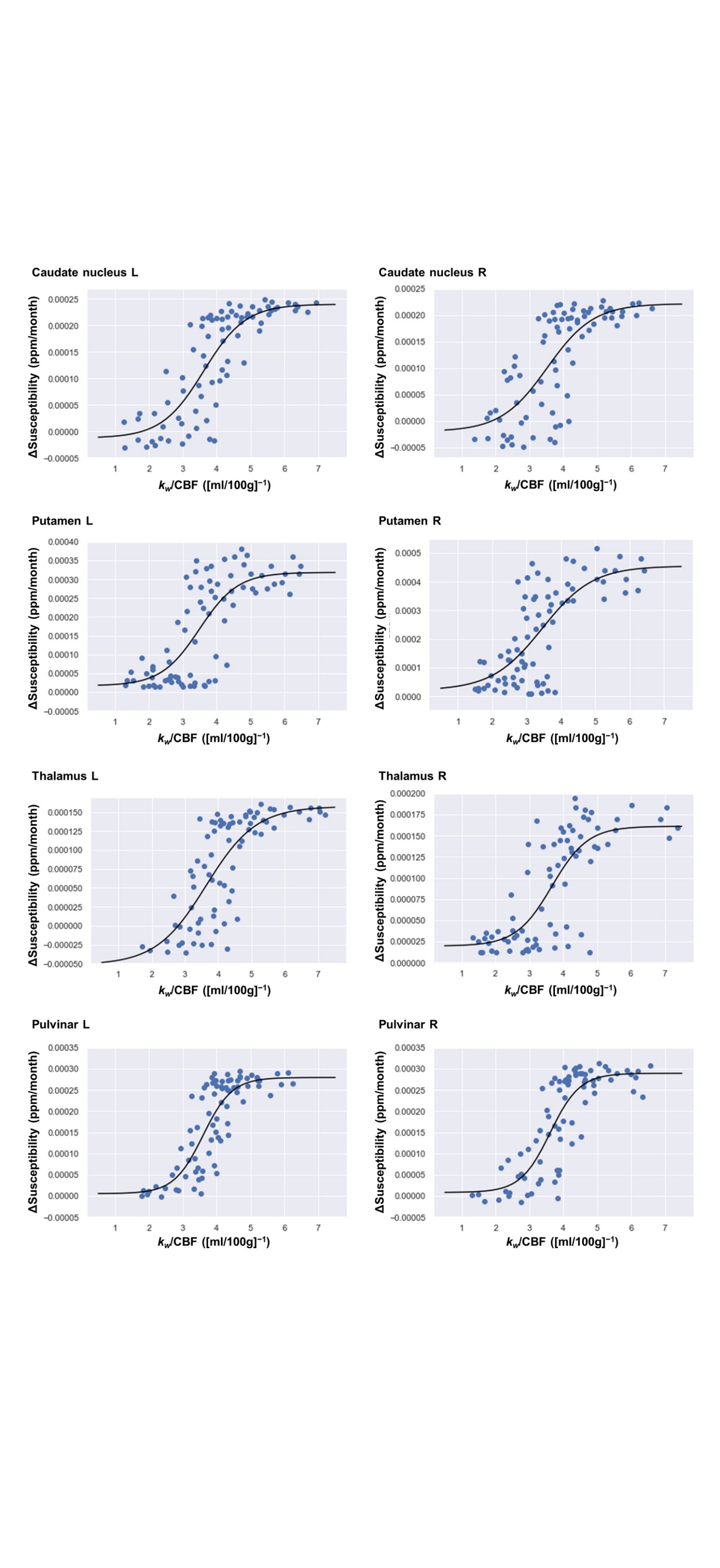
**and the sigmoidal curve-fitting models**


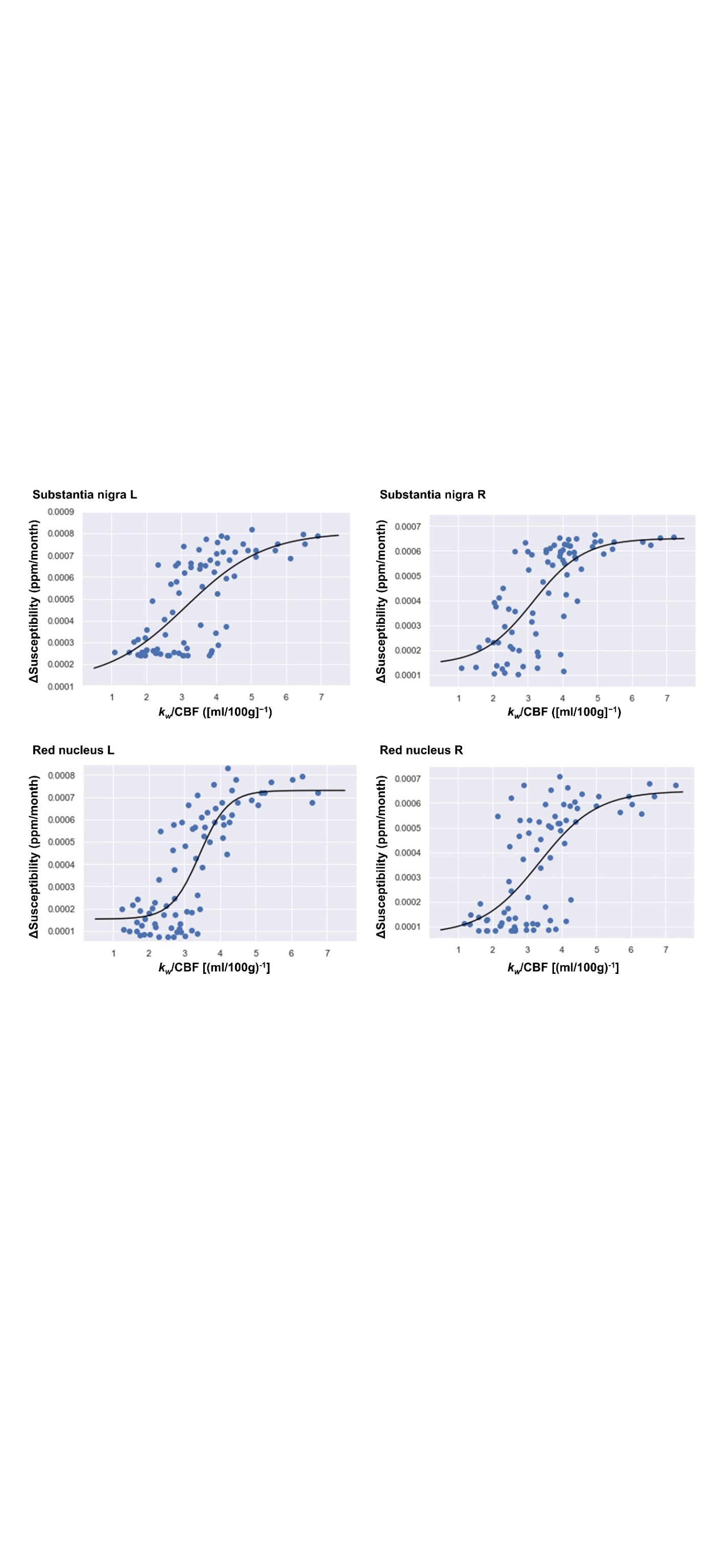
CBF = cerebral blood flow, L = left, R = right.
