# Supplementary material for: Relationship between brain iron dynamics and blood-brain barrier function during childhood: a quantitative magnetic resonance imaging study
Source: Fluids Barriers CNS. 2023 Aug 17;20:60. doi: 10.1186/s12987-023-00464-x (PMC10433620; doi:10.1186/s12987-023-00464-x)
Supplement: Supplementary file 2 — Additional file 2 [file 12987_2023_464_MOESM2_ESM.docx]

**Supplementary tables:**

**Table S1: Mean values from atlas-based analyses in all the subjects**

| Anatomical region | Susceptibility (× 10^−3^ ppm) | *k_w_*  (min^−1^) | *k_w_*/CBF ([mL/100g]^−1^) |
| --- | --- | --- | --- |
| Caudate nucleus L | 18.96 ± 8.37 | 83.90 ± 15.97 | 4.00 ± 1.32 |
| Caudate nucleus R | 18.25 ± 6.77 | 84.62 ± 15.96 | 3.81 ± 1.20 |
| Putamen L | 12.04 ± 7.35 | 87.55 ± 22.21 | 3.62 ± 1.30 |
| Putamen R | 12.36 ± 8.12 | 82.60 ± 19.80 | 3.43 ± 1.20 |
| Globus pallidus external L | 48.78 ± 35.36 | 75.99 ± 13.87 | 3.28 ± 1.22 |
| Globus pallidus external R | 51.32 ± 35.49 | 77.21 ± 14.23 | 3.20 ± 1.12 |
| Globus pallidus internal L | 48.59 ± 32.42 | 73.58 ± 16.06 | 3.15 ± 1.16 |
| Globus pallidus internal R | 49.56 ± 32.07 | 75.03 ± 15.57 | 3.09 ± 1.19 |
| Thalamus L | −1.82 ± 4.96 | 93.36 ± 12.16 | 4.28 ± 1.18 |
| Thalamus R | −0.46 ± 4.09 | 89.07 ± 17.37 | 3.68 ± 1.35 |
| Pulvinar L | 7.48 ± 10.90 | 91.38 ± 11.32 | 3.94 ± 0.96 |
| Pulvinar R | 9.02 ± 10.98 | 91.07 ± 14.86 | 3.98 ± 1.15 |
| Substantia nigra L | 33.67 ± 24.79 | 82.10 ± 17.08 | 3.41 ± 1.26 |
| Substantia nigra R | 35.57 ± 25.39 | 84.25 ± 17.09 | 3.56 ± 1.23 |
| Red nucleus L | 18.95 ± 16.14 | 80.84 ± 17.27 | 3.24 ± 1.24 |
| Red nucleus R | 19.38 ± 16.35 | 81.97 ± 19.53 | 3.37 ± 1.30 |

Note: Data are means ± standard deviation.

Abbreviation: CBF = cerebral blood flow, L = left, R = right.

**Table S2: Mean values from atlas-based analyses in the sedation-free subjects**

| Anatomical region | Susceptibility (× 10^−3^ ppm) | *k_w_*  (min^−1^) | *k_w_*/CBF ([mL/100g]^−1^) |
| --- | --- | --- | --- |
| Caudate nucleus L | 19.63 ± 8.46 | 84.41 ± 16.24 | 4.09 ± 1.40 |
| Caudate nucleus R | 18.81 ± 6.61 | 83.12 ± 15.45 | 3.76 ± 1.14 |
| Putamen L | 12.45 ± 7.49 | 85.89 ± 23.04 | 3.59 ± 1.28 |
| Putamen R | 12.64 ± 7.78 | 84.31 ± 20.29 | 3.61 ± 1.36 |
| Globus pallidus external L | 50.33 ± 33.41 | 76.35 ± 13.21 | 3.32 ± 1.28 |
| Globus pallidus external R | 52.14 ± 34.04 | 75.97 ± 13.78 | 3.11 ± 1.05 |
| Globus pallidus internal L | 49.22 ± 33.16 | 75.61 ± 17.32 | 3.07 ± 1.12 |
| Globus pallidus internal R | 50.34 ± 31.23 | 74.41 ± 14.23 | 3.18 ± 1.26 |
| Thalamus L | −1.43 ± 4.42 | 91.43 ± 12.01 | 4.08 ± 1.13 |
| Thalamus R | −0.38 ± 3.92 | 86.90 ± 15.02 | 3.55 ± 1.24 |
| Pulvinar L | 8.03 ± 10.12 | 90.12 ± 13.66 | 3.80 ± 0.92 |
| Pulvinar R | 9.14 ± 10.53 | 91.98 ± 15.05 | 4.03 ± 1.20 |
| Substantia nigra L | 35.14 ± 25.03 | 84.22 ± 17.08 | 3.59 ± 1.41 |
| Substantia nigra R | 37.09 ± 25.81 | 85.35 ± 18.23 | 3.62 ± 1.28 |
| Red nucleus L | 19.51 ± 15.89 | 81.47 ± 17.61 | 3.30 ± 1.28 |
| Red nucleus R | 19.86 ± 16.01 | 82.09 ± 18.72 | 3.41 ± 1.35 |

Note: Data are means ± standard deviation.

Abbreviation: CBF = cerebral blood flow, L = left, R = right.

**Table S3: Sensitivity analysis for *k_w_* values with varying ATT**

| Anatomical region | ATT = 1000 ms | ATT = 1400 ms | ATT = 1800 ms |
| --- | --- | --- | --- |
| Caudate nucleus L | 40.11 ± 8.86 | 61.23 ± 11.48 | 83.90 ± 15.97 |
| Caudate nucleus R | 41.15 ± 9.02 | 62.15 ± 10.89 | 84.62 ± 15.96 |
| Putamen L | 43.90 ± 11.41 | 66.78 ± 14.45 | 87.55 ± 22.21 |
| Putamen R | 40.93 ± 10.04 | 62.01 ± 13.19 | 82.60 ± 19.80 |
| Globus pallidus external L | 38.13 ± 7.45 | 54.62 ± 9.89 | 75.99 ± 13.87 |
| Globus pallidus external R | 39.32 ± 7.94 | 56.76 ± 10.13 | 77.21 ± 14.23 |
| Globus pallidus internal L | 37.47 ± 7.55 | 53.41 ± 9.14 | 73.58 ± 16.06 |
| Globus pallidus internal R | 38.87 ± 8.13 | 54.93 ± 8.79 | 75.03 ± 15.57 |
| Thalamus L | 53.08 ± 8.43 | 72.21 ± 10.48 | 93.36 ± 12.16 |
| Thalamus R | 51.45 ± 11.02 | 69.33 ± 14.41 | 89.07 ± 17.37 |
| Pulvinar L | 50.10 ± 7.65 | 70.45 ± 9.22 | 91.38 ± 11.32 |
| Pulvinar R | 49.24 ± 7.10 | 70.18 ± 8.94 | 91.07 ± 14.86 |
| Substantia nigra L | 44.30 ± 12.41 | 61.66 ± 15.03 | 82.10 ± 17.08 |
| Substantia nigra R | 46.62 ± 13.03 | 63.08 ± 15.48 | 84.25 ± 17.09 |
| Red nucleus L | 39.17 ± 10.67 | 58.49 ± 14.71 | 80.84 ± 17.27 |
| Red nucleus R | 40.24 ± 11.43 | 59.94 ± 15.20 | 81.97 ± 19.53 |

Note: Data are means ± standard deviation.

Abbreviation: CBF = cerebral blood flow, L = left, R = right.

**Table S4: Best-fitted regression model and derivative function of age**

| Anatomical region | Best-fitted regression model | Derivative function of age |
| --- | --- | --- |
| Caudate nucleus L | −7.89 × 10^−7^ × (age)^2^ + 2.53 × 10^−4^ × (age) + 7.52 × 10^−3^ | −1.58 × 10^−6^ × (age) + 2.53 × 10^−4^ |
| Caudate nucleus R | −7.76 × 10^−7^ × (age)^2^ + 2.31 × 10^−4^ × (age) + 8.35 × 10^−3^ | −1.55 × 10^−6^ × (age) + 2.31 × 10^−4^ |
| Putamen L | 6.27 × 10^−9^ × (age)^3^ − 2.66 × 10^−6^ × (age)^2^ + 3.91 × 10^−4^ × (age) − 8.23 × 10^−4^ | 1.89 × 10^−8^ × (age)^2^ − 5.32 × 10^−6^ × (age) + 3.91 × 10^−4^ |
| Putamen R | 1.29 × 10^−8^ × (age)^3^ − 4.50 × 10^−6^ × (age)^2^ + 5.34 × 10^−4^ × (age) − 2.91 × 10^−3^ | 3.86 × 10^−8^ × (age)^2^ − 9.00 × 10^−6^ × (age) + 5.34 × 10^−4^ |
| Globus pallidus external L | 1.61 × 10^−8^ × (age)^3^ − 7.56 × 10^−6^ × (age)^2^ + 1.42 × 10^−3^ × (age) − 8.68 × 10^−3^ | 4.833 × 10^-8^ × (age)^2^ − 1.5118 × 10^-5^ × (age) + 0.001424 |
| Globus pallidus external R | 1.71 × 10^−8^ × (age)^3^ − 8.22 × 10^−6^ × (age)^2^ + 1.51 × 10^−3^ × (age) − 7.27 × 10^−3^ | 5.127 × 10^-8^ × (age)^2^ − 1.6436 × 10^-5^ × (age) + 0.001507 |
| Globus pallidus internal L | 1.49 × 10^−8^ × (age)^3^ − 7.54 × 10^−6^ × (age)^2^ + 1.40 × 10^−3^ × (age) − 5.33 × 10^−3^ | 4.458 × 10^-8^ × (age)^2^ − 1.5078 × 10^-5^ × (age) + 0.001399 |
| Globus pallidus internal R | 2.56 × 10^−8^ × (age)^3^ − 1.09 × 10^−5^ × (age)^2^ + 1.67 × 10^−3^ × (age) − 7.78 × 10^−3^ | 7.692 × 10^-8^ × (age)^2^ − 2.1840 × 10^-5^ × (age) + 0.001672 |
| Thalamus L | −5.51 × 10^−7^ × (age)^2^ + 1.63 × 10^−4^ × (age) − 8.77 × 10^−3^ | −1.10 × 10^−6^ × (age) + 1.63 × 10^−4^ |
| Thalamus R | 3.66 × 10^−9^ × (age)^3^ − 1.44 × 10^−6^ × (age)^2^ + 2.00 × 10^−4^ × (age) − 6.92 × 10^−3^ | 1.10 × 10^−8^ × (age)^2^ − 2.88 × 10^−6^ × (age) + 2.00 × 10^−4^ |
| Pulvinar L | −8.34 × 10^−7^ × (age)^2^ + 2.98 × 10^−4^ × (age) − 6.90 × 10^−3^ | −1.68 × 10^−6^ × (age) + 2.98 × 10^−4^ |
| Pulvinar R | −9.19 × 10^−7^ × (age)^2^ + 3.16 × 10^−4^ × (age) − 5.90 × 10^−3^ | −1.84 × 10^−6^ × (age) + 3.16 × 10^−4^ |
| Substantia nigra L | 9.12 × 10^−9^ × (age)^3^ − 4.04 × 10^−6^ × (age)^2^ + 8.36 × 10^−4^ × (age) − 3.17 × 10^−3^ | 2.74 × 10^−8^ × (age)^2^ − 8.07 × 10^−6^ × (age) + 8.36 × 10^−4^ |
| Substantia nigra R | −1.58 × 10^−6^ × (age)^2^ + 6.71 × 10^−4^ × (age) + 5.73 × 10^−3^ | −3.17 × 10^−6^ × (age) + 6.71 × 10^−4^ |
| Red nucleus L | 1.75 × 10^−8^ × (age)^3^ − 6.42 × 10^−6^ × (age)^2^ + 8.56 × 10^−4^ × (age) − 8.79 × 10^−3^ | 5.26 × 10^−8^ × (age)^2^ − 1.29 × 10^−5^ × (age) + 8.56 × 10^−4^ |
| Red nucleus R | 1.08 × 10^−8^ × (age)^3^ − 4.57 × 10^−6^ × (age)^2^ + 7.25 × 10^−4^ × (age) − 6.64 × 10^−3^ | 3.25 × 10^−8^ × (age)^2^ − 9.14 × 10^−6^ × (age) + 7.25 × 10^−4^ |

Note: Age range was 2 months to 180 months.

Abbreviation: L = left, R = right.

**Table S5: Root-mean-square error of the fitting model**

|  | Root-Mean-Square Error (× 10^−3^) | |
| --- | --- | --- |
| Anatomical Region | Sigmoidal Curve-Fitting Model | Best-Fitted Regression Model* |
| Caudate nucleus L | 0.53 | 0.58 |
| Caudate nucleus R | 0.62 | 0.64 |
| Putamen L | 0.83 | 0.87 |
| Putamen R | 1.10 | 1.19 |
| Globus pallidus external L | 0.21 | 0.26 |
| Globus pallidus external R | 0.25 | 0.30 |
| Globus pallidus internal L | 0.27 | 0.30 |
| Globus pallidus internal R | 0.35 | 0.37 |
| Thalamus L | 0.43 | 0.46 |
| Thalamus R | 0.40 | 0.44 |
| Pulvinar L | 0.56 | 0.63 |
| Pulvinar R | 0.61 | 0.70 |
| Substantia nigra L | 1.46 | 1.49 |
| Substantia nigra R | 1.33 | 1.40 |
| Red nucleus L | 1.40 | 1.57 |
| Red nucleus R | 1.61 | 1.65 |

Note: Data are means ± standard deviation.

*Equations of the best-fitted regression models are listed in Supplemental Table 4.

Abbreviation: L = left, R = right.

**Table S6: Best-fitted regression model of scattergram between the Δsusceptibility and *k_w_*/CBF values**

| Anatomical region | Best-fitted regression model |
| --- | --- |
| Caudate nucleus L | −5.23 × 10^−6^ × (age)^3^ + 5.76 × 10^−5^ × (age)^2^ − 1.28 × 10^−4^ × (age) + 7.09 × 10^−5^ |
| Caudate nucleus R | −7.68 × 10^−6^ × (age)^2^ + 1.17 × 10^−4^ × (age) − 2.09 × 10^−4^ |
| Putamen L | −8.90 × 10^−6^ × (age)^3^ + 9.66 × 10^−5^ × (age)^2^ − 2.38 × 10^−4^ × (age) + 1.96 × 10^−4^ |
| Putamen R | −9.54 × 10^−6^ × (age)^3^ + 1.00 × 10^−4^ × (age)^2^ − 2.14 × 10^−4^ × (age) + 1.72 × 10^−4^ |
| Globus pallidus external L | 2.56 × 10^−5^ × (age)^4^ − 4.17 × 10^−4^ × (age)^3^ + 2.32 × 10^−3^ × (age)^2^ − 4.85 × 10^−3^ × (age) + 3.61 × 10^−3^ |
| Globus pallidus external R | 2.75 × 10^−5^ × (age)^4^ − 4.48 × 10^−4^ × (age)^3^ + 2.49 × 10^−3^ × (age)^2^ − 5.21 × 10^−3^ × (age) + 3.79 × 10^−3^ |
| Globus pallidus internal L | 1.74 × 10^−5^ × (age)^4^ − 2.85 × 10^−4^ × (age)^3^ + 1.57 × 10^−3^ × (age)^2^ − 3.09 × 10^−3^ × (age) + 2.12 × 10^−3^ |
| Globus pallidus internal R | −5.27 × 10^−5^ × (age)^2^ + 7.19 × 10^−4^ × (age) − 8.78 × 10^−4^ |
| Thalamus L | −2.97 × 10^−6^ × (age)^3^ + 3.25 × 10^−5^ × (age)^2^ − 5.76 × 10^−5^ × (age) − 2.51 × 10^−5^ |
| Thalamus R | −2.03 × 10^−6^ × (age)^3^ + 2.03 × 10^−5^ × (age)^2^ − 1.83 × 10^−5^ × (age) − 1.76 × 10^−5^ |
| Pulvinar L | −1.28 × 10^−5^ × (age)^3^ + 1.40 × 10^−4^ × (age)^2^ − 3.81 × 10^−4^ × (age) + 3.09 × 10^−4^ |
| Pulvinar R | −1.06 × 10^−5^ × (age)^3^ + 1.15 × 10^−4^ × (age)^2^ − 2.94 × 10^−4^ × (age) + 2.14 × 10^−4^ |
| Substantia nigra L | −1.06 × 10^−5^ × (age)^3^ + 1.11 × 10^−4^ × (age)^2^ − 2.27 × 10^−4^ × (age) + 2.99 × 10^−4^ |
| Substantia nigra R | −1.86 × 10^−5^ × (age)^2^ + 2.55 × 10^−4^ × (age) − 2.06 × 10^−4^ |
| Red nucleus L | −1.97 × 10^−5^ × (age)^3^ + 2.13 × 10^−4^ × (age)^2^ − 5.21 × 10^−4^ × (age) + 5.05 × 10^−4^ |
| Red nucleus R | −1.21 × 10^−5^ × (age)^2^ + 2.11 × 10^−4^ × (age) − 2.03 × 10^−4^ |

Note: Age range was 2 months to 180 months.

Abbreviation: CBF = cerebral blood flow, L = left, R = right.

**Table S7: Inflection point of sigmoidal curve-fitting model**

| Anatomical region | Inflection point ([mL/100g]^−1^) |
| --- | --- |
| Caudate nucleus L | 3.50 |
| Caudate nucleus R | 3.53 |
| Putamen L | 3.48 |
| Putamen R | 3.46 |
| Globus pallidus external L | 2.99 |
| Globus pallidus external R | 2.96 |
| Globus pallidus internal L | 3.03 |
| Globus pallidus internal R | 3.01 |
| Thalamus L | 3.64 |
| Thalamus R | 3.67 |
| Pulvinar L | 3.59 |
| Pulvinar R | 3.60 |
| Substantia nigra L | 3.12 |
| Substantia nigra R | 3.15 |
| Red nucleus L | 3.42 |
| Red nucleus R | 3.35 |

Abbreviation: L = left, R = right.
